# Targeting P-glycoprotein: Investigation of piperine analogs for overcoming drug resistance in cancer

**DOI:** 10.1038/s41598-017-08062-2

**Published:** 2017-08-11

**Authors:** Safiulla Basha Syed, Hemant Arya, I-Hsuan Fu, Teng-Kuang Yeh, Latha Periyasamy, Hsing-Pang Hsieh, Mohane Selvaraj Coumar

**Affiliations:** 1 Centre for Bioinformatics, School of Life Sciences, Pondicherry University, Kalapet, Puducherry 605014 India; 2 DBT-Interdisciplinary Program in Life Sciences, Pondicherry University, Kalapet, Puducherry 605014 India; 3 Institute of Biotechnology and Pharmaceutical Research, National Health Research Institutes, 35 Keyan Road, Zhunan, Miaoli County 350 Taiwan, ROC; 4 Department of Biochemistry & Molecular Biology, School of Life Sciences, Pondicherry University, Kalapet, Puducherry 605014 India; 5 Department of Chemistry, National Tsing Hua University, Hsinchu, 350 Taiwan, ROC

## Abstract

P-glycoprotein (P-gp) is a drug transporter that effluxes chemotherapeutic drugs and is implicated in the development of resistance of cancer cells to chemotherapeutic drugs. To date, no drug has been approved to inhibit P-gp and restore chemotherapy efficacy. Moreover, majority of the reported inhibitors have high molecular weight and complex structures, making it difficult to understand the basic structural requirement for P-gp inhibition. In this study, two structurally simple, low molecular weight piperine analogs Pip1 and Pip2 were designed and found to better interact with P-gp than piperine *in silico*. A one step, acid-amine coupling reaction between piperic acid and 6,7-dimethoxytetrahydroisoquinoline or 2-(3,4-dimethoxyphenyl)ethylamine afforded Pip1 and Pip2, respectively. *In vitro* testing in drug resistant P-gp overexpressing KB (cervical) and SW480 (colon) cancer cells showed that both analogs, when co-administered with vincristine, colchicine or paclitaxel were able to reverse the resistance. Moreover, accumulation of P-gp substrate (rhodamine 123) in the resistant cells, a result of alteration of the P-gp efflux, was also observed. These investigations suggest that the natural product analog – Pip1 ((2*E*,4*E*)-5-(benzo[*d*][1,3]dioxol-5-yl)-1-(6,7-dimethoxy-3,4-dihydroisoquinolin-2(1 *H*)-yl)penta-2,4-dien-1-one) – is superior to piperine and could inhibit P-gp function. Further studies are required to explore the full potential of Pip1 in treating drug resistant cancer.

## Introduction

Cancer is a leading causes of death in the world today^1^. Chemotherapy, along with surgery and radiation therapy, constitutes the main treatment option available for many types of cancer, but multi-drug resistance (MDR) is a major problem^2–7^. Among the various modalities of drug resistance, overexpression of membrane transporters such as P-glycoprotein (MDR1), multi-drug resistance-associated protein 1 (MRP1), breast cancer resistance protein (BCRP) which efflux the chemotherapeutic drugs out of the cancer cell plays a significant role in the failure of chemotherapy^8–11^.

P-glycoprotein (P-gp/*MDR1*/ABCB1) is a 170 KD membrane protein belonging to the ATP-binding cassette super family and a major drug transporter reported to be overexpressed in different cancers^12^. The use of many anti-cancer drugs such as vincristine, vinblastine, docetaxel, cyclophosphamide, flutamide, ifosfamide and paclitaxel; all substrates of P-gp, induce expression of the *MDR1* gene, leading to MDR^13^. However, inhibition of P-gp efflux function has been shown to reverse the MDR of cancer^14–17^. A number of P-gp inhibitors have been tested in clinical trials, but to date none of have been approved for human use^18^.

First generation P-gp inhibitors, including verapamil and cyclosporine A, were not specifically synthesized to inhibit P-gp; and were also found to interact with other transporters, resulting in an unpredictable pharmacokinetics^7, 19^. The second generation inhibitors biricodar, valspodar, and dexverapamil were less toxic and more potent than the first generation inhibitors, but were found to interact with drug metabolizing enzymes such as CYP450 3A4, thereby affecting the metabolism of other chemotherapeutic drugs^16^. The third generation inhibitors tariquidar, elacridar and zosuquidar showed potent P-gp inhibition and acceptable toxicity profiles in phase I clinical trials^20–22^, but phase III studies of tariquidar in combination with carboplatin/paclitaxel or with vinorelbine had to be closed early, for toxicity reasons^20^. More recently, tetrandrine (CBT-1^®^; NSC-77037), a bis-benzylisoquinoline alkaloid isolated from *Stephania tetrandra* and found to modulate the P-gp inhibitory activity^23^, was submitted to phase I clinical trials (www.ClinicalTrials.gov; NCT03002805) to investigate its utility in the treatment of drug resistant cancers, in combination with doxorubicin.

Detailed structure-activity relationship (SAR) and quantitative structure-activity relationship (QSAR) studies of P-gp inhibitors have concluded that the compounds with high lipophilicity (logP) tend to be better P-gp inhibitors^24–26^. Moreover, the P-gp inhibitors entered into clinical trials have an average logP value of more than five (calculated using MarvinSketch v5.6.2), and an average molecular weight of >500 Da (supplementary result Table S1). Lipophilicity is one of the most important physicochemical properties used in hit/lead/drug candidate selection^27, 28^, and previous studies have suggested that compounds with high lipophilicity have an increased likelihood of toxic events^29–31^. Moreover, compounds with high molecular weight and high lipophilicity have a higher probability of being discontinued from clinical development^32^. Therefore, the identification of compounds with potent P-gp inhibitory activity and low molecular weight and lipophilicity is imperative, as they should have a lower chance of failure in the drug discovery pipeline.

On the other hand, more than 25% of the drugs used today are derived from plant origin; while other 25% are chemically altered natural products. It is reported that 60% of the anticancer drugs approved since 1940 are of natural origin. Some of the plant based anticancer drugs which are in clinical use/trials are vinca alkaloids (vincristine, vinblastine), taxanes (taxol, docetaxel), podophyllotoxins (etoposide, teniposide), and camptothecins (topotecan, irinotecan)^33^. Knowing the anticancer properties of these natural products, many research groups have tested them either alone or in combination with other chemotherapeutic drugs to explore their P-gp inhibitory activities in P-gp overexpressing cancer cell lines^34–36^. Subsequently, structure-activity relationship studies of some of the natural compounds were carried out to shed light on the importance of the functional groups in the natural compounds for P-gp inhibitory activities^34^.

Piperine, an alkaloid and a major ingredient of black pepper (*Piper nigrum*) and long pepper (*Piper longum*)^37^, has been reported to impart P-gp inhibitory activity, as well as antitumor, antioxidant, antimicrobial, bio-enhancer and hepatoprotective activity^38^. Due to its low molecular weight and simple structure, piperine could serve as a lead molecule for the design of potent P-gp inhibitors. Thus, the aim of this study is to investigate the P-gp inhibitory activity of piperine analogs of lower molecular weight and lower lipophilicity than previous known P-gp inhibitors.

**Figure 1:**
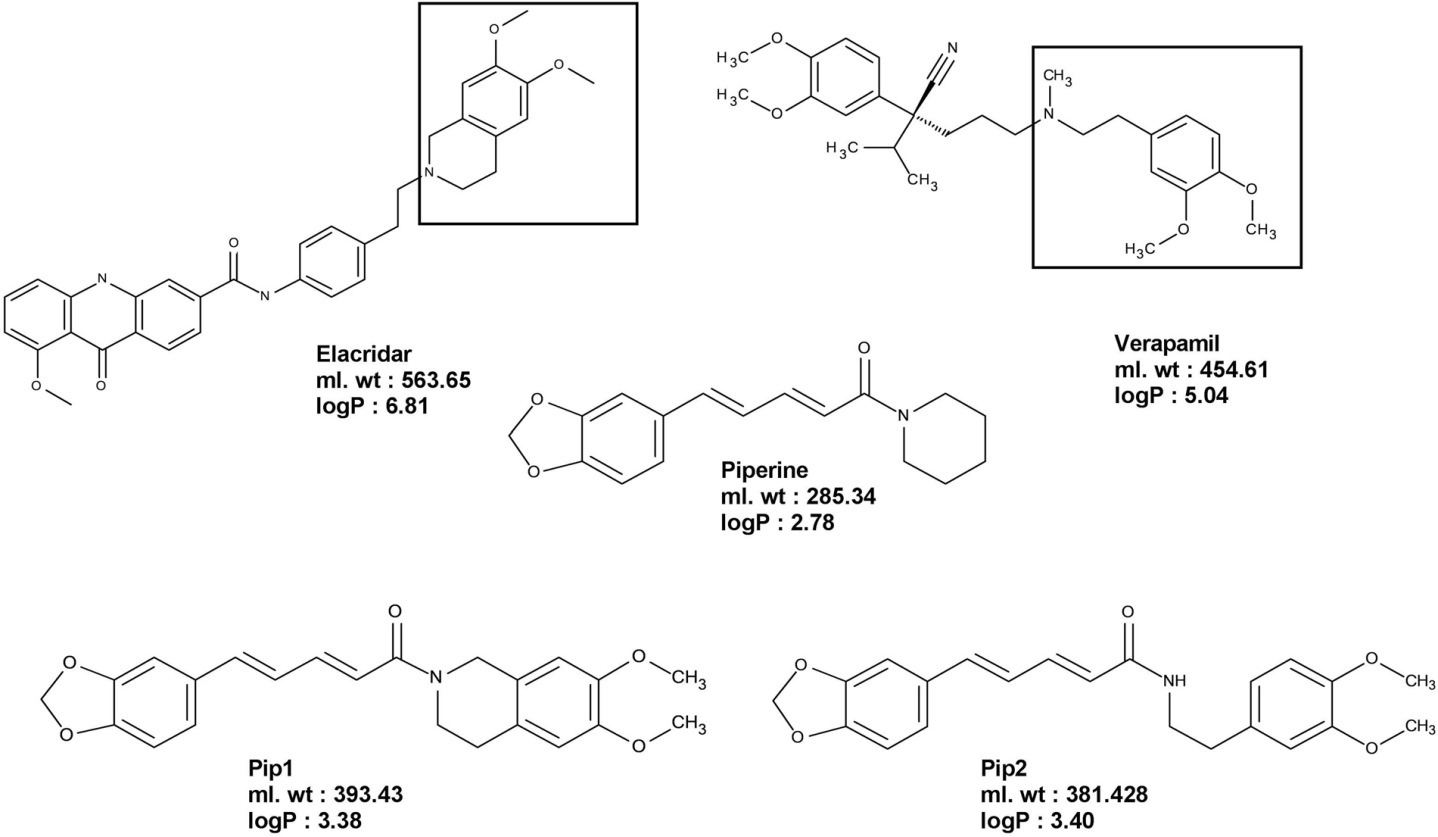
Conceptual design of piperine analogs – Pip1 and Pip2 – as P-gp inhibitors.

Two small piperine analogs Pip1 (ml.wt: 393.43) and Pip2 (ml.wt: 381.42) were designed based on the SAR information garnered from reported P-gp inhibitors^34^. Since many phytochemical alkaloids with benzodioxol ring were reported to modulate the P-gp activity^34^, modification of piperine at the piperidine ring side was envisaged. Therefore, in Pip1, the piperidine ring has been replaced with a 6,7-dimethoxytetrahydroisoquinoline a moiety, which is widely present in modulators of P-gp including the third generation P-gp inhibitors (tariquidar and elacridar) (Fig. 1)^39^. This moiety is also present in the alkaloid tetrandrine, currently in clinical trials. Introduction of this moiety only slightly increased the lipophilicity of Pip1 (logP = 3.38), as compared to piperine (logP = 2.78). Wang *et al*., have previously reported that a highly effective P-gp modulator should possess a logP value of 2.92 or higher^24^.

In Pip2 (logP = 3.40), the piperidine ring has been replaced with 2-(3,4-dimethoxyphenyl)ethylamine, a moiety present in the first generation P-gp inhibitor verapamil (Fig. 1). This moiety can be considered as a ring-opened analog of 6,7-dimethoxytetrahydroisoquinoline.

**Figure 2:**
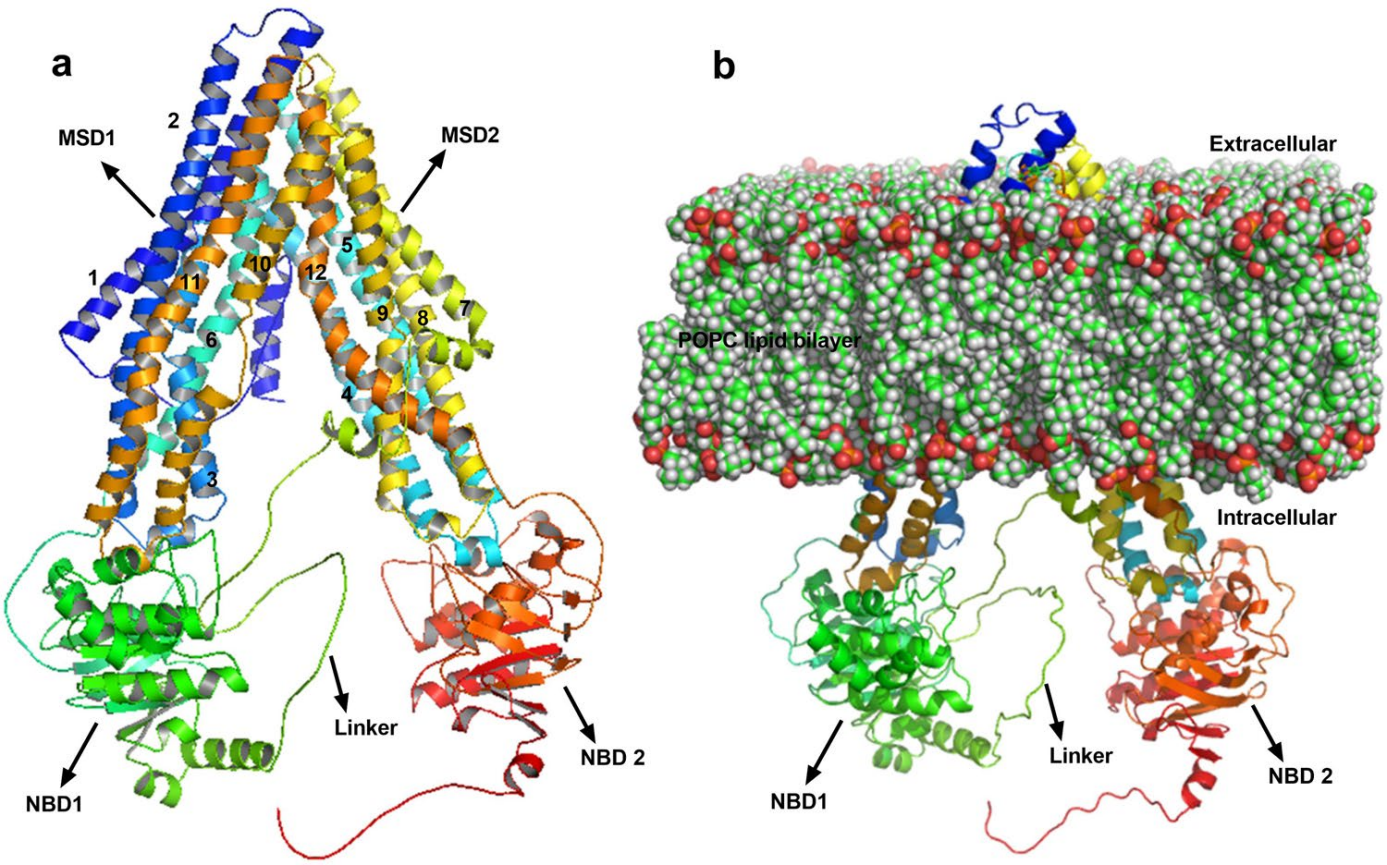
Molecular modeling of human P-gp structure. (**a**) 3D Structure of human P-gp model build by homology modeling using Modeller software. PDB ID: 4F4C is used as template (MSD: membrane spaning domain; NBD: nucleotide binding domain); Transmembrane (TM) helices are denoted by numbers from 1 to 12. (**b**) Human P-gp model embedded in the POPC lipid bilayer, for molecular dynamics simulation; prepared using the CHARMM-GUI server.

Initially, *in silico* molecular docking and molecular dynamic simulation (MD) studies were carried out using the human P-gp (hP-gp) homology model (Fig. 2) to understand the binding of the two analogs with P-gp. Based on the promising *in silico* docking and MD simulation results, both analogs were synthesized and tested in two different cancer cell lines overexpressing P-gp. The results suggest that piperine analogs at a concentration of 2 μM were more potent than piperine at increasing the sensitivity of P-gp overexpressing KB and SW480 cancer cells to chemotherapeutic drugs vincristine (VCR), colchicine (COL) and paclitaxel (PTX), without affecting the parental KB and SW480 cells. In particular, Pip1 at a concentration of 2 μM exerted a 9.7, 7.06 and 6.29-fold reversal of VCR, COL and PTX resistance in drug resistant KB cervical cancer cells, respectively. This was found to be more or less equivalent to the standard P-gp inhibitor verapamil which caused a 12.4, 4.1, and 4.57-fold reversal of VCR, COL and PTX resistance in P-gp overexpressing KB cells, respectively. Moreover, both the analogs increased the accumulation of rhodamine 123 in P-gp overexpressing KB and SW480 drug resistant cells, suggesting that the increase in the efficacy of chemotherapeutic drugs is due to the inhibitory activity of these analogs on P-gp. Further studies are required to explore the full potentiality of Pip1 in treating drug resistant cancer. Moreover, due to its small structure and potent P-gp inhibitory activity, pip1 is proposed as a lead molecule to further design potent P-gp inhibitors with low lipophilicity and potent P-gp inhibitory activity.

## Results

### *In silico* investigation of piperine analogs

#### Homology modeling of human P-gp

The crystal structure of *C. elegans* (PDB ID: 4F4C, chain A) with a sequence identity of 46% to hP-gp, was selected as template to model the target hP-gp. The similarities in functional properties and amino acid sequences between *C. elegans* and hP-gp suggested that the structure of *C. elegans* P-gp would be a reasonable starting point for structural studies of hP-gp^40^. The sequence alignment of *C. elegans* and hP-gp obtained from ClustalW shows fine alignment of conserved residues in nucleotide binding domain (NBD) and transmembrane domain (TM) regions (supplementary result Fig. S1). A total of 100 models were generated using MODELLER 9v12 software and the model with the least DOPE (−148463.03) and molpdf score (7120.67) was selected for further validation by ProCheck. The Ramachandran plot (supplementary result Fig. S2) of hP-gp homology model (Fig. 2a) showed 92.5% of residues (1064 amino acids) in the most favored region; 6.6% of residues (76 amino acids) in additional allowed region; 0.9% of residues in generously allowed region; and no residues in the disallowed region. The RMSD between template (PDB ID: 4F4C) and the modeled hP-gp was found to be 2.68 Å, suggesting that the modeled human P-gp protein had significant structural similarities with *C. elegans* P-gp. Based on the Ramachandran plot and RMSD value (supplementary result Fig. S2), the selected model was deemed satisfactory and used for further molecular docking and molecular dynamics simulation studies.

#### Molecular docking investigations of piperine analogs with P-gp

**Figure 3:**
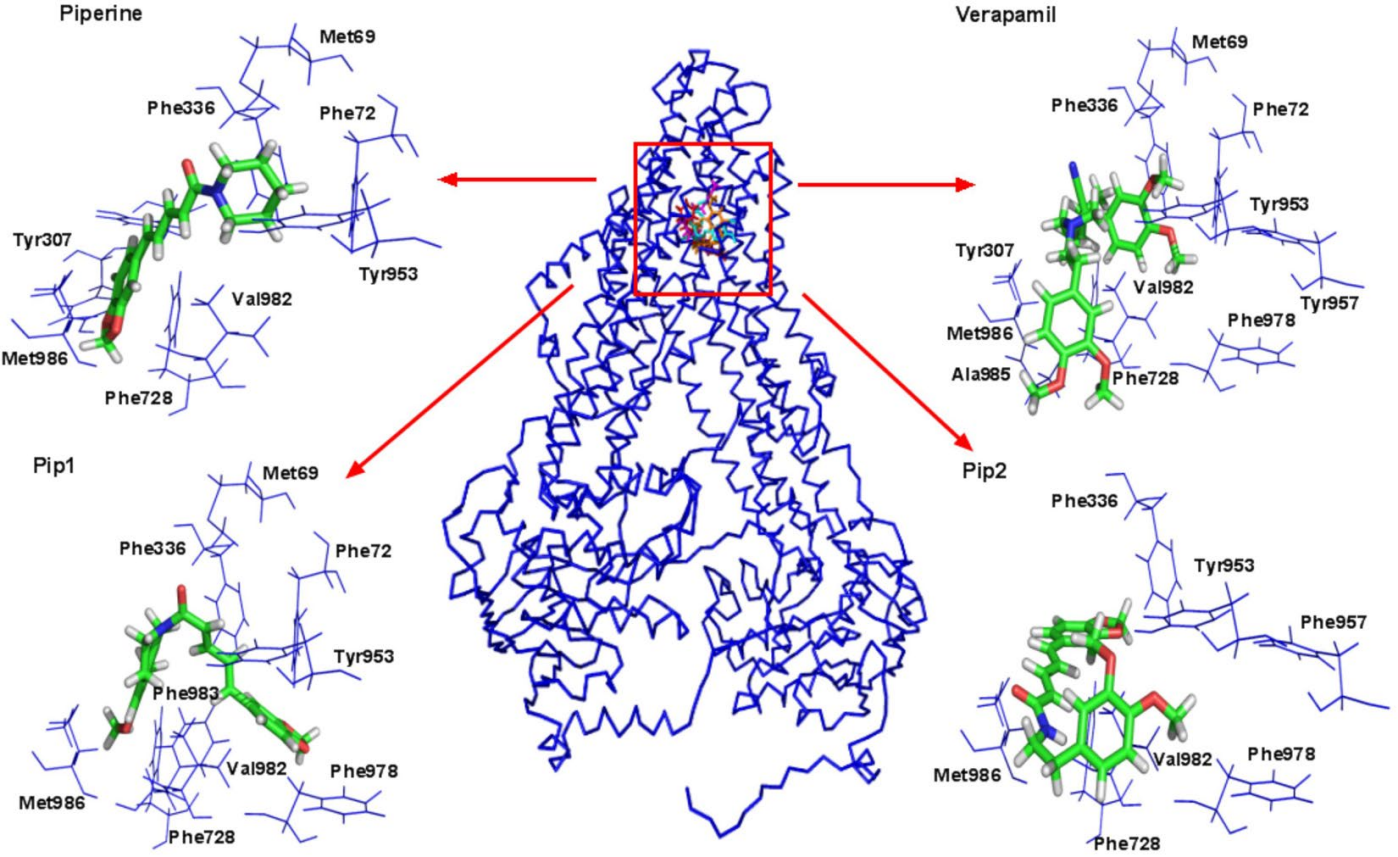
3D interactions of human P-gp with piperine, verapamil and piperine analogs (Pip1 and Pip2) predicted by docking studies conducted using Glide XP software.

In this study, the modeled P-gp was used to perform docking studies with piperine analogs (Pip1 and Pip2) using glide XP software. Verapamil and piperine were used as reference compounds to compare the molecular interactions of piperine analogs with the modeled hP-gp. The drug binding site for the docking was defined based on the previously reported studies: residues Leu65, Met69, Phe72 (TM1), Thr199 (TM3), Ser222 (TM4), Ile306, Tyr307 (TM5), Phe336, Leu339, Ile340, Ala342, Phe343 (TM6), Phe728 (TM7), Ala841 (TM9), Ile868, Gly872 (TM10), Phe942, Thr945, Tyr953, Phe957 (TM11), Leu975, Phe978, Val981, Val982, Phe983, Gly984, Ala985 and Met986 (TM12). A cut-off distance of 3 and 5 Å were used to determine the presence of hydrogen bonds and hydrophobic interactions respectively, between the protein residues and the ligands. Piperine and verapamil were able to interact with the drug binding site residues with glide scores of −7.82 and −7.34 and glide energies of −26.85 and −39.50 kcal mol^−1^, respectively. Based on the Prime MM/GBSA approach, the free energy of binding (ΔG) of piperine and verapamil to the modeled hP-gp was estimated to be −59.72 and −97.41 kcal mol^−1^, respectively (Table 1). Piperine was interacted by forming an H-bond with Tyr307 and hydrophobic interactions with Met69, Phe72, Phe336, Leu339, Phe728, Tyr953, Val982, Phe983 and Met986 (Fig. 3). Verapamil interacted with the important residues of hP-gp via a π-π interaction (to Phe336) and hydrophobic interactions (to Met69, Phe72, Phe336, Leu339, Ile340, Phe728, Ile868, Tyr953, Phe957, Phe978, Val981, Val982, Phe983, Ala985 and Met986) (Fig. 3). Pip1 was found to interact with the same key residues that were found to be interacting with verapamil; e.g., π-π interactions (Phe72 and Phe983) and hydrophobic interactions (Met69, Phe336, Leu339, Phe728, Tyr953, Phe978, Val982, Phe983 and Met986) (Fig. 3). Pip1 interacted with P-gp with a glide score of −7.99, a binding free energy of −56.75 kcal mol^−1^, and a glide energy of −26.06 kcal mol^−1^ (Table 1). Similarly, Pip2 was also found to interact with the hydrophobic amino acid residues (Met69, Phe336, Leu339, Phe728, Ile868, Tyr953, Phe957, Phe978, Val981, Val982, Phe983, Ala985 and Met986) (Fig. 3) with a glide score of −8.16, a binding free energy of −74.95 kcal mol^−1^, and a glide energy of −35.29 kcal mol^−1^ (Table 1). Recently, Kim *et al*., carried out a molecular docking study in which around 500 binding poses of verapamil in P-gp were checked. The residues that interacted with the ligand over 300 times out of the 500 binding poses were considered to be the probable verapamil binding site residues^41^. In our docking study, both the designed piperine analogs were found to be encapsulated by these important residues. Considering the glide scores, binding free energies, glide energies, and hydrophobic interactions, Pip1 and Pip2 were found to be more promising than piperine. To confirm that both were able to interact with P-gp in a stable manner, the P-gp-piperine analogs complexes were subjected to MD studies.

**Table 1:** Docking analysis of P-gp with verapamil, piperine and piperine analogs (Pip1 and Pip2).

S. No	Compound	Glide G-Score	Glide energy (kcal mol^−1)^	ΔG binding free energy (kcal mol^−1^)	Interacting residues
1	Verapamil	−7.34	−39.50	−97.41	Phe336 (π-π interaction); Met69, Phe72, Phe336, Leu339, Ile340, Phe728, Ile868, Tyr953, Phe957, Phe978, Val981, Val982, Phe983, Ala985 and Met986 (hydrophobic interaction)
2	Piperine	−7.82	−26.85	−59.72	Tyr307 (Hydrogen bond); Met69, Phe72, Phe336, Leu339, Phe728, Tyr953, Val982, Phe983 and Met986 (hydrophobic interaction)
3	Pip1	−7.99	−26.06	−56.75	Phe72 and Phe983 (π-π interaction); Met69, Phe336, Leu339, Phe728, Tyr953, Phe978, Val982, Phe983 and Met986 (hydrophobic interaction)
4	Pip2	−8.16	−35.29	−74.95	Met69, Phe336, Leu339, Phe728, Ile868, Tyr953, Phe957, Phe978, Val981, Val982, Phe983, Ala985 and Met986 (hydrophobic interaction)

#### Molecular dynamics simulation of P-gp-piperine analogs complex

Based on the docking results, the docked protein-ligand complexes (P-gp-piperine, P-gp-verapamil, P-gp-Pip1 and P-gp-Pip2) were selected for a 50 ns molecular dynamics (MD) simulation study using GROMACS v5.0.4, to check their stability. In MD simulation experiments, physical movement of atoms and molecules are allowed for a short time (nanoseconds). The forces between atoms and molecules, and their potential energy were determined by using force field parameters^42, 43^. Since P-gp is a membrane bound protein, it was embedded in a layer of lipid membrane and then subjected to MD simulation. For this purpose, protein-ligand complexes as well as P-gp alone were submitted to the CHARMM-GUI web server, a platform to generate protein-ligand-lipid membrane complex systems for MD simulation. The output files (Fig. 2b) were downloaded, further equilibrated, and finally a MD production run for 50 ns was carried out on GROMACS v5.0.4. The stability of the system was evaluated by calculating the backbone root mean square deviations (RMSDs) and root mean square fluctuations (RMSFs) of individual amino acid residues of the modeled hP-gp with respect to the initial structure. Interactions between the ligand and drug binding residues were evaluated and the potential energy (PE) was calculated.

**Figure 4:**
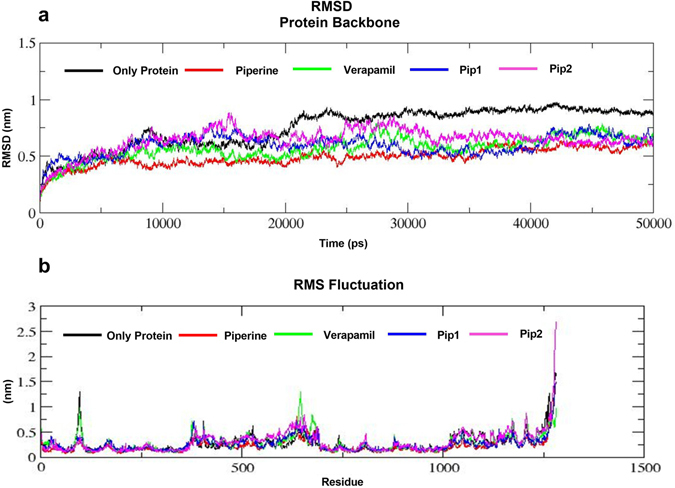
Molecular dynamics (MD) simulation (50 ns) of verapamil, piperine, and piperine analogs (Pip1, Pip2) in complex with the human P-gp model. (**a**) Protein backbone root mean square deviation (RMSD) graph, and (**b**) individual amino acid root mean square fluctuation (RMSF) graph.

RMSD graphs of the protein alone (apo form) and protein-ligand complex showed that an equilibrium condition was reached within 200 ps of the simulation period. The protein backbone RMSD varied between 0.6–0.9 nm (P-gp alone), 0.4–0.6 nm (P-gp-piperine complex), 0.5–0.65 nm (P-gp-verapamil complex), 0.6–0.65 nm (P-gp-Pip2 complex) and 0.5–0.6 nm (P-gp-Pip1 complex). The average RMSD of final 20 ns (30 to 50 ns) was found to be 0.89 nm for P-gp alone, and between 0.55 to 0.67 nm for P-gp-piperine, P-gp-verapamil, P-gp-Pip1 and P-gp-Pip2 complexes (Fig. 4a). RMSD analysis showed that throughout the MD simulation period, the backbone of all the protein-ligand complexes were highly stable compared to the protein alone complex, suggesting that presence of a ligand stabilized the complex. Analysis of the RMSF graph showed that the residues in the loop and the terminal region were highly flexible; however most of the key drug binding residues (Met69, Phe72, Tyr307, Phe336, Leu339, Ile340, Phe343, Phe728, Ile868, Tyr953, Phe957, Phe978, Val981, Val982, Phe983, Ala985 and Met986) were less flexible in the protein-ligand complexes, as compared to the apo form (Fig. 4b; supplementary result Table S2). Furthermore, the initial (docked pose) and the final (after 50 ns MD simulation) protein-ligand complex structures were extracted and analyzed to determine if the hydrophobic interactions were maintained throughout the simulations. It was found that most of the drug binding residues maintained hydrophobic interaction with piperine and its analogs. However, some of the residues lost interactions and few other residues which didn’t show interactions in the docked pose were found to interact with piperine and its analogs at the end of the simulation (supplementary result Table S3). From this, we can conclude that the protein-ligand complexes were stable and maintained hydrophobic interactions with the drug binding residues. The potential energies of protein-ligand complexes were calculated; it was found that the designed analogs-P-gp complex had a lower potential energy than the reference compounds: −2.1080e + 06 kJmol^−1^ (P-gp-piperine complex), −2.09956e + 06 kJmol^−1^ (P-gp-verapamil complex), −2.16192e + 06 kJmol^−1^ (P-gp-Pip1 complex), and −2.14651e + 06 kJmol^−1^ (P-gp-Pip2 complex). By analyzing RMSD, RMSF, potential energy and hydrophobic interactions in this way, the protein-ligand complexes (piperine analogs) were determined to be stable.

Overall, the molecular docking and molecular dynamics simulation studies suggest that the designed analogs (Pip1 and Pip2) could interact with the drug binding site of the hP-gp protein in a manner very similar to that of the reference compounds, piperine and verapamil. Accordingly, their synthesis and testing as P-gp inhibitors was pursued.

### Synthesis of piperine analogs

**Figure 5:**
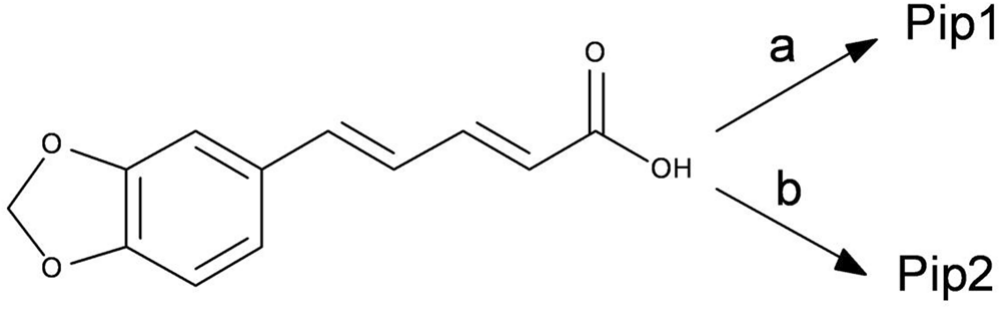
Synthesis of piperine analogs. (**a**) 6,7-Dimethoxy-1,2,3,4-tetrahydro-isoquinoline, isobutyl chloroformate, N-methylmorpholine, THF, 0 °C to rt, 25%. (**b**) 2-(3,4-Dimethoxy-phenyl)-ethylamine, isobutyl chloroformate, N-methylmorpholine, THF, 0 °C to rt, 51%.

For the preparation of piperine analogs, piperic acid was reacted with isobutyl chloroformate to generate the carbonic anhydride intermediate. The intermediate was converted *in situ* to the desired amide products – Pip1 and Pip2 – by reacting with 6,7-dimethoxy-1,2,3,4-tetrahydro-isoquinoline and 2-(3,4-dimethoxy-phenyl)-ethylamine, respectively (Fig. 5). The final products were characterized using ^1^H NMR and LC-MS, and their purity was determined by HPLC (supplementary result Figs.  S4–S9). The ^1^H NMR spectrum for Pip1 showed three broad peaks at *δ* 2.85, 3.79 and 4.72, corresponding to the three –CH_2_– groups of tetrahydro-isoquinoline ring. Pip2 showed two proton quartets at *δ* 3.60 and two proton triplets at *δ* 2.82 for the two –CH_2_– groups of phenyl-ethylamine side chain and a broad peak for one proton (NH) at *δ* 5.48. Mass spectral data confirms the structure of the synthesized piperine derivatives.

### *In vitro* investigation of piperine analogs

#### Piperine analogs increased the sensitivity of resistant KB Ch^R^ 8–5 cells to vincristine, colchicine and paclitaxel

**Figure 6:**
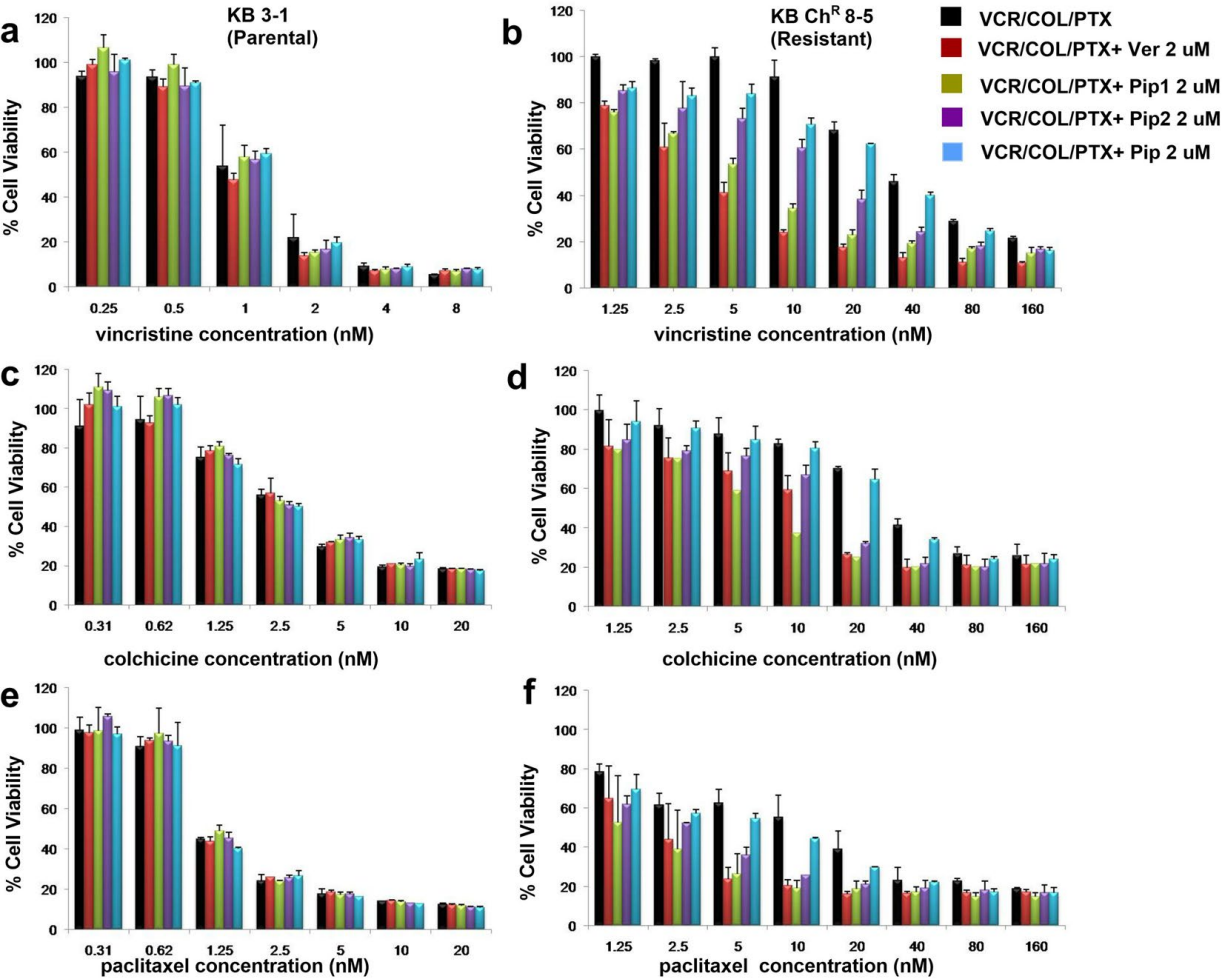
Cytotoxicity of vincristine (VCR) in (**a**) KB 3–1 (parental), and (**b**) KB Ch^R^ 8–5 (resistant); colchicine (COL) in (**c**) KB 3–1 (parental), and (**d**) KB Ch^R^ 8–5; paclitaxel (PTX) in (**e**) KB-3–1 (parental), and (**f**) KB Ch^R^ 8–5 cells. The cells were treated either with VCR/COL/PTX alone or in combination with Verapamil (Ver), Pip1, Pip2 or piperine (Pip) (the concentration of Ver, Pip, Pip1 and Pip2 were chosen at which the cell viability was more than 95% both in KB 3–1 and KB Ch^R^ 8–5 cell lines). Values with error bars represent the mean ± SEM of two or three independent experiments, each done in triplicates.

The resistant cancer cell line KB Ch^R^ 8–5 was derived from the drug sensitive parent cell line KB 3–1^44^. These cells exhibited lowered sensitivity to vincristine (VCR), colchicine (COL) and paclitaxel (PTX) treatment. The IC_50_ value of VCR, COL and PTX in KB Ch^R^ 8–5 was 36.08, 31.99, and 6.17 nM respectively, compared to the parental KB 3–1 cells having IC_50_ value of 0.86, 2.88, and 1.27 nM for VCR, COL and PTX, respectively. This suggests that there is a 42, 11 and 5-fold decreased sensitivity for VCR, COL and PTX in resistant KB cells, respectively (Fig. 6, Table 2). Interestingly, a 72 hr co-treatment of resistant KB cells with VCR/COL/PTX along with Pip1 (2 μM) or Pip2 (2 μM) resulted in a 9.7 or 3-fold reversal of VCR resistance, with a significant change in the IC_50_ of VCR to 3.70 nM (p < 0.005) and 11.47 nM (p < 0.05); 7 and 2.9-fold reversal of COL resistance with a significant change in the IC_50_ of COL to 4.53 nM (p < 0.05) and 11.13 nM; 6.3 and 3.7-fold reversal of PTX resistance with a significant change in the IC_50_ of PTX to 0.98 (p < 0.05) and 1.66 (p < 0.05), respectively (Table 2 and supplementary result Fig. S10). There was no significant change in the IC_50_ of VCR/COL/PTX in drug sensitive parental KB cells upon co-treatment with piperine analogs Pip1 or Pip2 (IC_50_, VCR 0.66 and 0.74 nM; COL 2.93 and 2.77 nM; PTX 1.42 and 1.37 nM). Co-treatment with verapamil, a known P-gp inhibitor, caused a 12.4, 4.1 and 4.6-fold reversal of VCR, COL and PTX resistance in resistant KB cells respectively, with a significant change in the IC_50_ of VCR to 2.89 nM (p < 0.005), IC_50_ of COL to 7.78 nM (p < 0.05) and IC_50_ of PTX to 1.35 nM (p < 0.05) (Table 2 and supplementary result Fig. S10). Whereas, co-treatment did not altered the sensitivity in parental KB cells (IC_50_ for VCR 0.54 nM, COL 2.95 nM and PTX 1.32 nM) (Fig. 6 and Table 2). On the other hand, co-treatment of VCR/COL/PTX with piperine did not significantly alter the IC_50_ of VCR/COL/PTX in both resistant and parental KB cells (Fig. 6, Table 2 and supplementary result Fig. S10).

**Table 2:** The effect of piperine analogs on the reversal of resistance in KB 3-1 (parental) and KB Ch^R^ 8-5 (resistant) cell lines on co-treatment with VCR/COL/PTX.

Treatment	IC_50_ (nM)	Fold resistance	IC_50_ (nM)	Fold resistance (FR)	Fold resistance reversal (FRR)
	KB 3-1 (Parental)		KB Ch^R^ 8-5 (Resistant)		
**Vincristine (VCR)**	**0.86** ± **0.24**	**1**	**36.08** ± **6.08**	**41.95**	**0**
VCR+ Ver (2 µM)	0.54 ± 0.15	0.62	2.89 ± 0.89**	3.36	12.4
VCR+ Pip (2 µM)	0.74 ± 0.12	0.86	31.72 ± 1.72	36.88	1.1
VCR+ Pip1 (2 µM)	0.66 ± 0.16	0.76	3.70 ± 0.70**	4.3	9.7
VCR+ Pip2 (2 µM)	0.74 ± 0.18	0.86	11.47 ± 1.47 *	13.33	3.1
**Colchicine (COL)**	**2.88** ± **0.95**	**1**	**31.99 ± 9.46**	**11.1**	**0**
COL+ Ver (2 µM)	2.95 ± 0.69	1.02	7.78 ± 2.70*	2.7	4.1
COL+ Pip (2 µM)	2.56 **±** 0.69	0.88	26.75 ± 7.69	9.28	1.19
COL+ Pip1 (2 µM)	2.93 ± 0.92	1.01	4.53 ± 0.88*	1.57	7.06
COL+ Pip2 (2 µM)	2.77 ± 0.84	0.96	11.13 ± 3.4	3.86	2.87
**Paclitaxel (PTX)**	**1.27** ± **0.41**	**1**	**6.17** ± **2.07**	**4.85**	**0**
PTX+ Ver (2 µM)	1.32 ± 0.43	1.03	1.35 ± 0.41*	1.06	4.57
PTX + Pip (2 µM)	1.27 ± 0.42	1	3.50 ± 0.68	2.75	1.76
PTX + Pip1 (2 µM)	1.42 ± 0.54	1.11	0.98 ± 0.38*	0.77	6.29
PTX + Pip2 (2 µM)	1.37 ± 0.46	1.07	1.66 ± 0.19*	1.3	3.71

*p < 0.05 and **p < 0.005 versus VCR/COL/PTX treatment alone.

#### Piperine analogs increased the sensitivity of resistant colorectal adenocarcinoma cell line SW480-VCR to vincristine and paclitaxel

**Figure 7:**
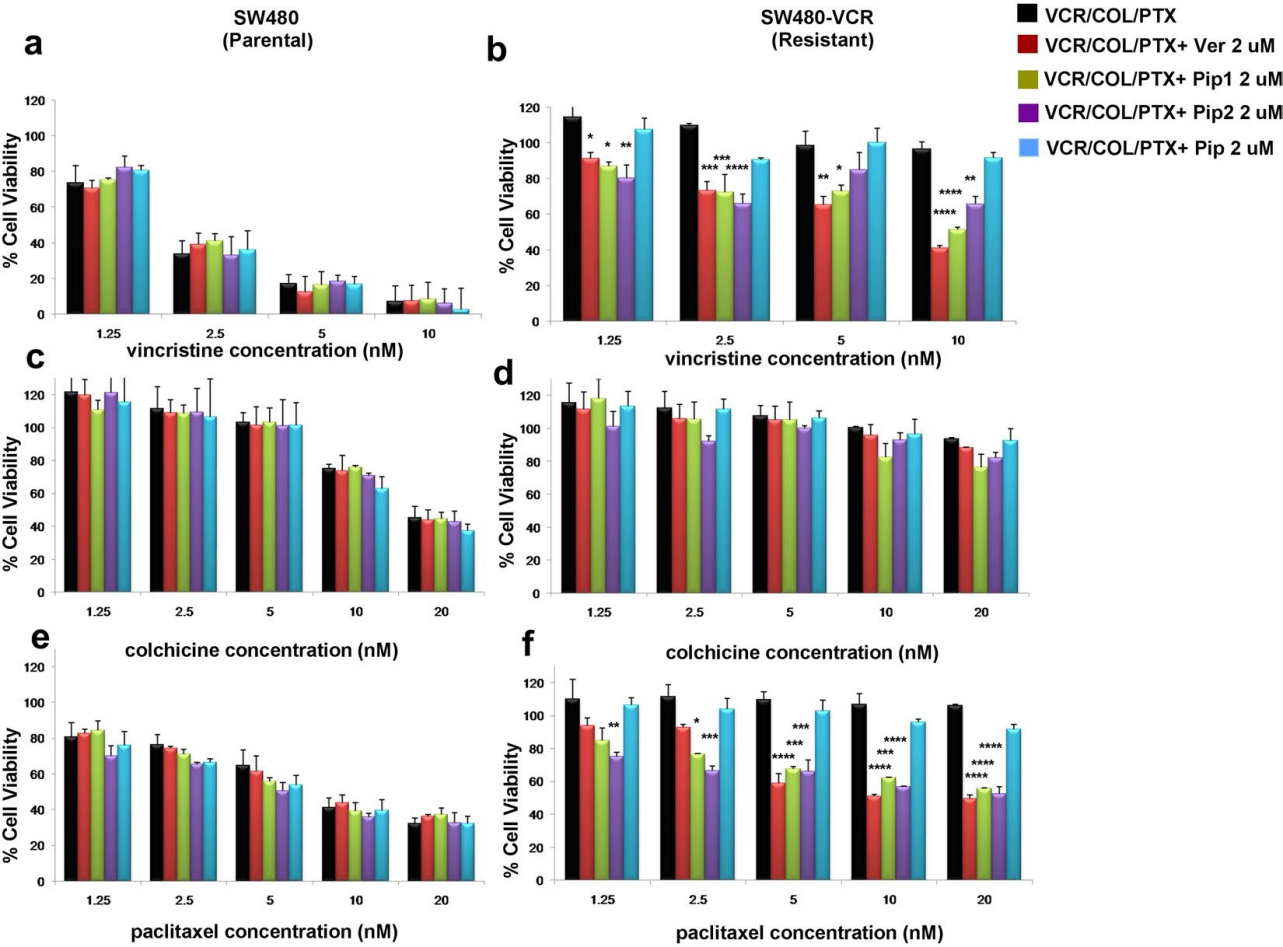
Cytotoxicity of vincristine (VCR) in (**a**) SW480 (parental), and (**b**) SW480-VCR (resistant); colchicine (COL) in (**c**) SW480 (parental), and (**d**) SW480-VCR (resistant); paclitaxel (PTX) in (**e**) SW480 (parental), and (**f**) SW480-VCR (resistant) cells. The cells were treated either with VCR/COL/PTX alone or in combination with Verapamil (Ver), Pip1, Pip2 or piperine (Pip). Values with error bars represent the mean ± SEM of two independent experiments, each done in triplicates. * p < 0.05 and ** p < 0.005, *** p < 0.0005 and **** p < 0.0001.

The resistance reversal activity of piperine analogs was further tested in resistant SW480 colorectal adenocarcinoma cell line (SW480-VCR), derived from the drug sensitive parent cell line SW480 by continuous exposure to VCR. The parent SW480 cells were found to be very sensitive to VCR treatment with cell viability at 10 nM of VCR was <10%; whereas, the resistant SW480 cells were unaffected at the same concentration of VCR (100% cell viability) and the cell viability was found to be very significant (p < 0.0001) compared to the parental SW480 cells (supplementary result Fig. S11). A 72 hr co-treatment of resistant SW480 with VCR along with Pip1 (2 μM) or Pip2 (2 μM) or verapamil resulted in a significant reduction (p < 0.0001, p < 0.005, and p < 0.0001) in the cell viability of resistant SW480 cells (≈50% cell viability at 10 nM VCR), without affecting the cell viability of parental SW480 cells (Fig. 7). Similarly, at 10 nM of PTX the cell viability of parental SW480 cells was <50%; whereas, the resistant SW480 cell viability was around 100%, suggesting that the resistant SW480-VCR cells were significantly (p < 0.0005) resistant to PTX treatment as well (supplementary result Fig. S11). Interestingly, a 72 hr co-treatment of resistant SW480-VCR with PTX along with 2 μM of Pip1, Pip2 or verapamil resulted in significant reversal (p < 0.0001) of resistance and the cell viability was reverted back to 50% (Fig. 7). Whereas, no significant change in the cell viability was observed in the resistant SW480 cells co-treated with PTX and piperine. Interestingly, parental SW480 cells were not sensitive to COL (10 nM, 75% cell viability) as compared to VCR (10 nM, 7% cell viability) or PTX (10 nM, 41% cell viability). Moreover, no significant difference in the cell viability of parental and resistant SW480 cells treated with COL (10 nM) was observed (supplementary result Fig. S11). Therefore, no difference was found in the cell viability of both parental and resistant SW480 cells co-treated with COL along with Pip1, Pip2, Pip and verapamil (Fig. 7).

These results suggest that piperine analogs (Pip1 and Pip2) could effectively reverse the resistance of chemotherapeutic drugs in the resistant KB (KB Ch^R^ 8–5) and SW480 (SW480-VCR) cells, as compared to piperine. Moreover, Pip1 was as potent as verapamil in reversing the drug resistance.

#### Piperine analogs decreased the colony forming ability of resistant KB Ch^R ^8–5 cells when co-treated with vincristine

**Figure 8:**
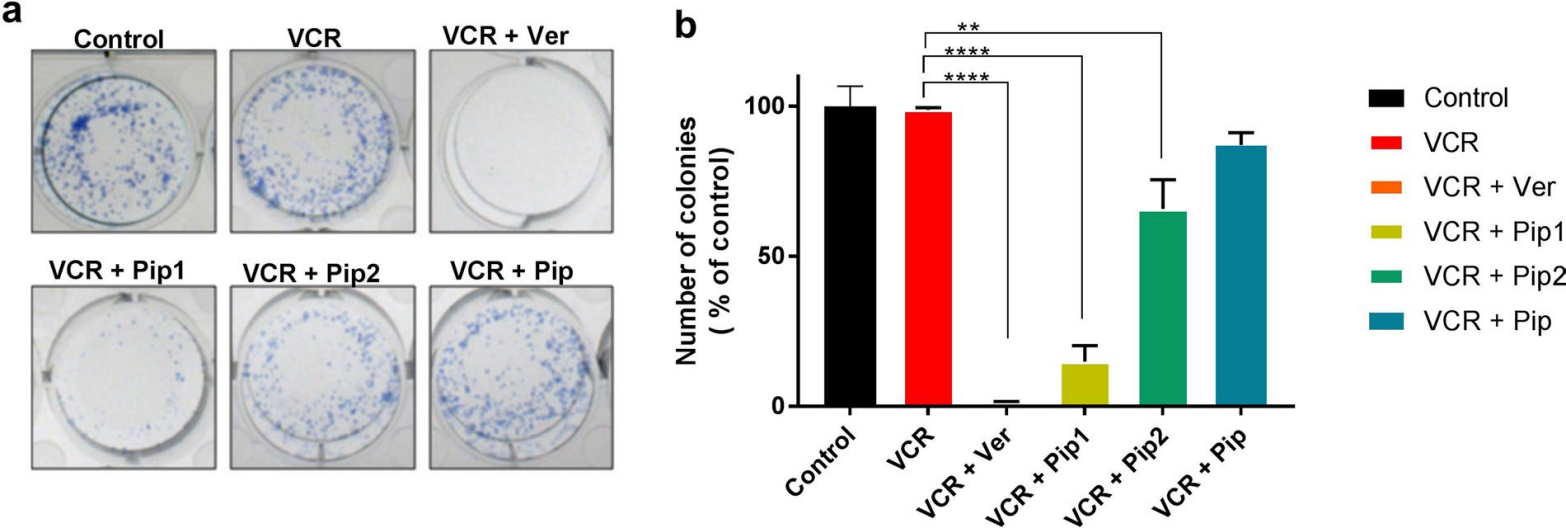
Colony forming ability of resistant KB Ch^R^ 8–5 cells. (**a**) The cells were treated with vincristine (VCR; 5 nM) alone or in combination with 2 μM each of verapamil (Ver), piperine (Pip), Pip1, and Pip2 for 72 h. Cells incubated only with culture media served as a control. (**b**) Quantification of number of colonies formed, as a percentage of control treatment. Error bars represent standard error of mean (SEM) of three independent experiments. **p < 0.05 and ****p < 0.0001 versus VCR 5 nM alone.

As the KB Ch^R^ 8–5 cells were more resistant to VCR, the effect of drug co-treatment on the colony forming ability of resistant KB cells was studied with different concentrations of VCR (5–40 nM), in combination with a fixed concentration (2 μM) of verapamil, piperine and piperine analogs. The colony forming ability of resistant KB cells was significantly reduced when they were co-treated with vincristine and piperine analogs, as compared to vincristine treatment alone. Co-treatment with vincristine (5 nM) and Pip1 (2 μM) or verapamil (2 μM) was more significant (p < 0.0001) than that of treatment with vincristine alone (5 nM) (Fig. 8) and also found to be significant (p < 0.05) in comparison with the cells treated with higher concentration of vincristine alone (40 nM) (supplementary result Fig. S12). Moreover, no colonies were observed when cells were co-treated with vincristine (10 nM) and Pip1 (2 μM) or verapamil (2 μM) (Fig. 8). Similarly, there was a significant (p < 0.005) reduction in the colony formation of the cells treated with a combination of vincristine 5 nM and Pip2 compared to the cells treated with vincristine alone 5 nM. In contrast, co-treatment with a combination of vincristine (5–40 nM) and piperine (2 μM) had a smaller effect on colony formation which was found to be insignificant (Fig. 8). However, there was no change in colony forming ability between the control cells which received only culture media and cells treated with piperine analogs, verapamil or piperine alone. The results suggest that the piperine analogs, particularly Pip1, when co-treated with vincristine effectively abrogate the colony forming ability of resistant KB cells.

#### Overexpression of P-gp in resistant KB Ch^R^ 8–5 and SW480-VCR cells

To elucidate the possible reason for drug resistance, both the parental (KB and SW480) and resistant (KB Ch^R^ 8–5 and SW480-VCR) cells were checked for the P-gp expression by western blot analysis using mouse monoclonal antibody, which binds to 1040–1280 amino acids of P-gp of human origin^45^. The results showed that there was a significant overexpression of P-gp in drug resistant KB Ch^R^ 8–5 and SW480-VCR cells, compared to the parental KB and SW480 cells (supplementary result Fig. S13) and could be the major reason for the observed drug resistance^44^.

#### P-gp localizes to the plasma membrane in resistant KB Ch^R^ 8–5 and SW480-VCR cells

**Figure 9:**
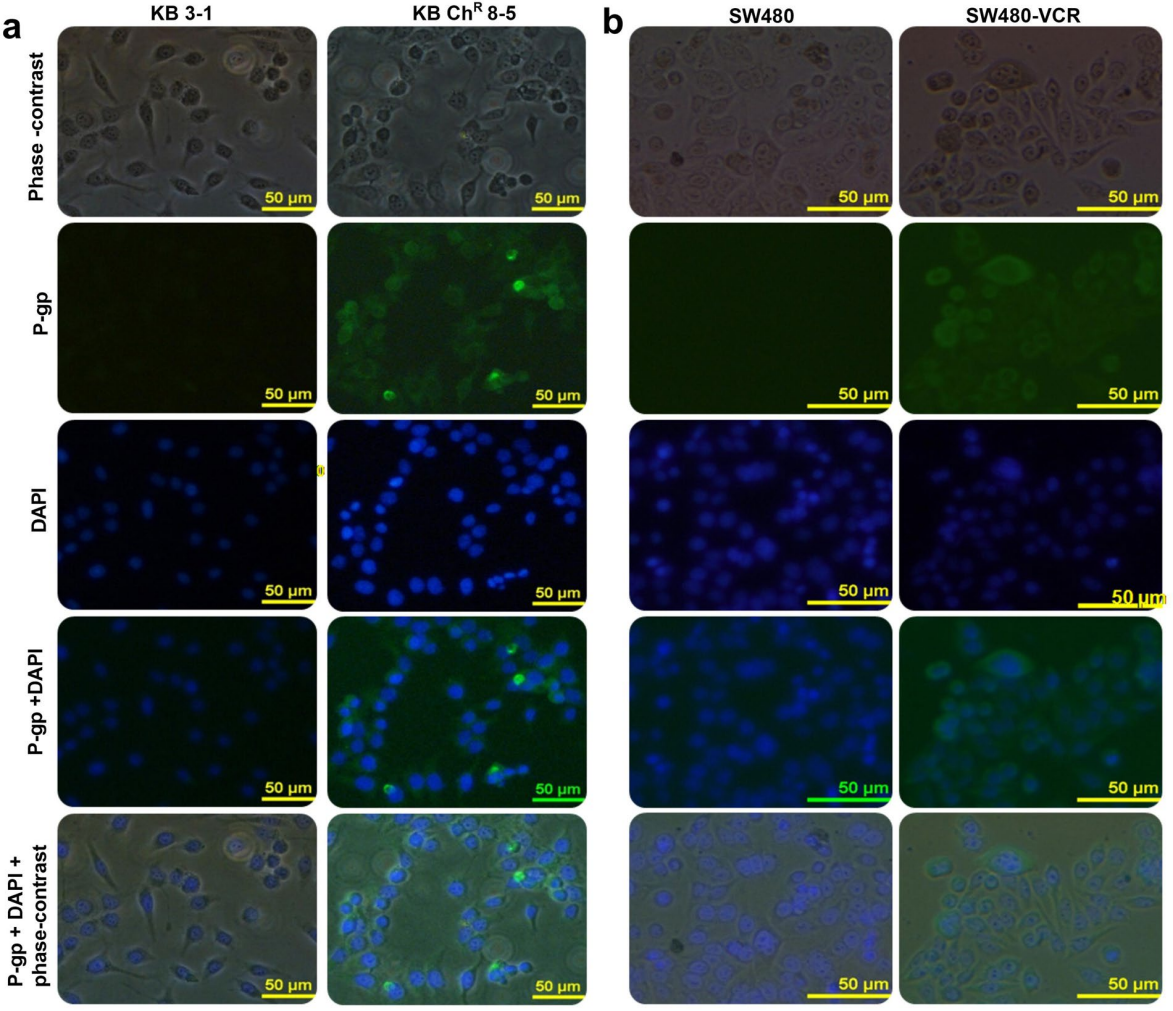
P-gp localization by immunofluorescence microscopy in (**a**) parental KB 3–1 and resistant KB Ch^R^ 8–5 cells and (**b**) parental SW480 and resistant SW480-VCR cells. The phase contrast, P-gp (green) and DAPI nuclear counter stain (blue). High levels of P-gp protein was localized to the plasma membrane of resistant cell line, KB Ch^R^ 8–5 and SW480-VCR cells.

The sub-cellular localization of P-gp plays a major role in determining the concentration of P-gp substrates inside the cells; P-gp localized to the plasma membrane directly effluxes the cytotoxic drugs out of the cells and thereby reduces their cellular concentration^46^. Accordingly, immunofluorescent detection of P-gp was carried out to characterize the sub-cellular localization of P-gp. High levels of P-gp at the plasma membrane of resistant KB Ch^R^ 8–5 and SW480-VCR cells were observed compared to the parental KB and SW480 cells (Fig. 9), suggesting that the P-gp could actively transport vincristine out of the cells, leading to the observed resistance.

#### Piperine analogs increased rhodamine 123 accumulation in resistant KB Ch^R^ 8–5 and SW480-VCR cells

**Figure 10:**
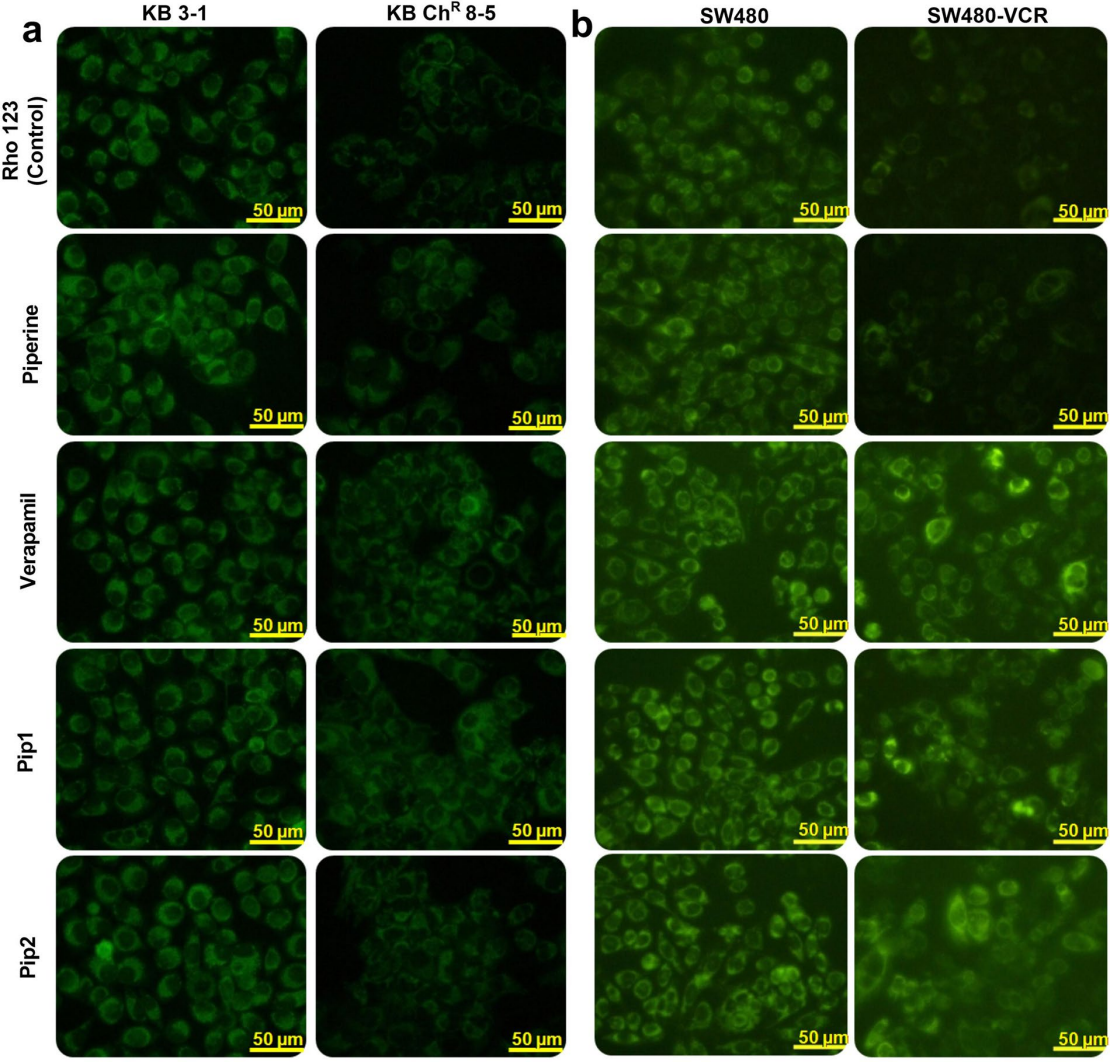
Effect of piperine analogs on Rho 123 accumulation in (**a**) KB 3–1 (parental) and KB Ch^R^ 8–5 (resistant) cells and (**b**) SW480 (parental) and SW480-VCR (resistant) cells. Cells were incubated with 4 μM of verapamil, piperine, Pip1, and Pip2. The compounds concentration in this assay was twice as that used for the cell viability and clonogenic assay, so as to clearly differentiate the Rho 123 accumulation between the control and treated cells. All the images were acquired using 20 × objective in a Nikon Eclipse Ti-S inverted fluorescence microscope (Scale 100 µM) (Nikon Instruments, Melville, New York), and processed with Nikon Br 4.0 Software.

The fluorescent dye rhodamine 123 (Rho123) is a well-known reference P-gp substrate used to characterize the P-gp inhibitory potential of drugs^47^. Rho123 accumulation in both the parental cell line (KB and SW480) and the resistant cell line (KB Ch^R^ 8–5 and SW480-VCR) were assessed, by treating them with and without the piperine analogs. A Rho123 accumulation assay showed that both the resistant cell lines treated with verapamil (4 µM) or Pip1 (4 µM) had increased Rho123 accumulation, as compared to the untreated (control) cells which were incubated with only Rho123 (10 µM) (Fig. 10). Moreover, the accumulation of Rho123 in resistant cells treated with Pip1 (4 µM) was almost equal to the cells treated with verapamil (4 µM). However, treatment with Pip2 (4 µM) resulted in lower levels of Rho123 accumulation in KB Ch^R^ 8–5 cells but similar level of accumulation in SW480-VCR, as compared to the treatment with verapamil or Pip1. There was no significant difference in Rho123 accumulation between the untreated and piperine (4 µM) treated resistant cells (supplementary result Fig. S14). On the other hand, similar amounts of Rho123 accumulation were observed in both the parental KB and SW480 cells, with and without the drug treatment (Fig. 10).

The accumulation of Rho123 only in the resistant cells by piperine analogs suggest that they could modulate the P-gp functional activity, thereby reducing the P-gp mediated efflux of Rho123 in the resistant KB Ch^R^ 8–5 and SW480-VCR cells. The increased sensitivity of resistant cells to VCR, COL and PTX when co-treated with piperine analogs, coupled with increased accumulation of Rho123 on treatment with piperine analogs, suggest that the reversal of resistance is due to the decreased efflux of VCR, COL and PTX by altered P-gp function.

#### Piperine analogs increased apoptosis in resistant KB Ch^R^ 8–5 cells when co-treated with vincristine

**Figure 11:**
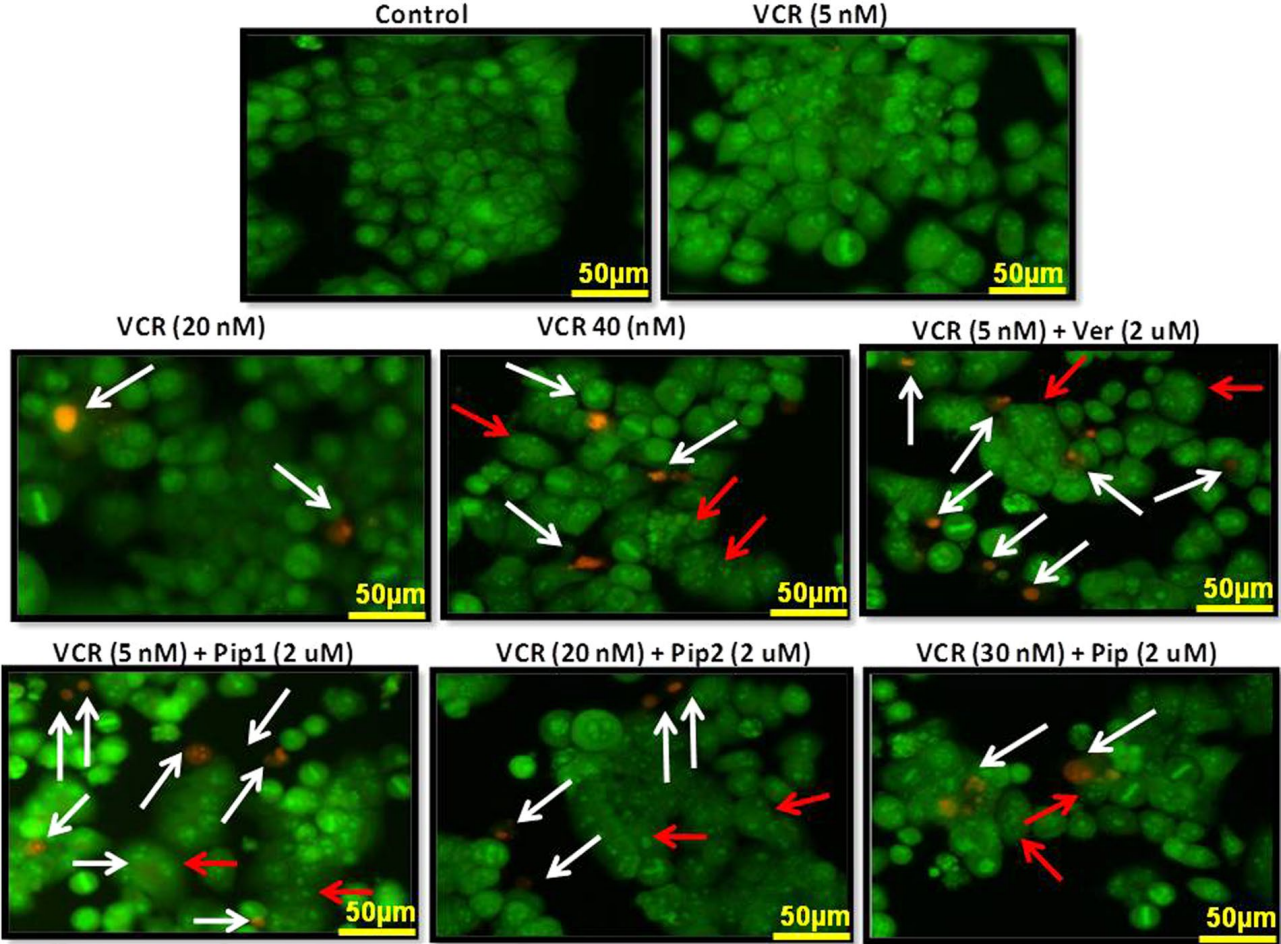
Evaluation of apoptosis incidence by acridine orange/ethidium bromide (AO/EB) dual staining. KB Ch^R^ 8–5 cells were treated either with vincristine alone or combination of vincristine with piperine (Pip), verapamil (Ver), Pip1, or Pip2; control cells were treated with the culture media containing drug vehicle (DMSO). The apoptotic cells (orange to red fluorescence nuclei) are indicated by white arrows, whereas the red arrows indicate the nuclear fragmentation. All the images were acquired using 20 × objective in a Nikon Eclipse Ti-S inverted Fluorescence microscope (Nikon Instruments, Melville, New York) and the images were processed with Nikon Br 4.0 Software.

Vincristine resistant KB cells were incubated with a fixed concentration of drug combination, i.e., vincristine 5 nM + verapamil 2 µM, vincristine 5 nM + Pip1 2 µM, vincristine 20 nM + Pip2 2 µM, and vincristine 30 nM + piperine 2 µM for 24 h (concentrations were chosen based on the clonogenic assay), to check the apoptosis incidence. Acridine orange is an intercalating agent which is taken up by both viable and non viable cells and emits green fluorescence upon binding to double stranded DNA; whereas, red fluorescence when bound to a single stranded DNA or RNA. Ethidium bromide is also an intercalating agent; however, it is taken up only by the nonviable cells and emits red fluorescence upon binding to DNA^48^. As shown in the Fig. 11, cells treated with either vincristine 5 nM + verapamil 2 µM or vincristine 5 nM + pip1 2 µM had an almost equal incidence of late apoptosis, showing orange to red fluorescence nuclei with nuclear fragmentation, compared to the cells treated with a higher concentration of vincristine (40 nM) alone. Similar results were observed in the cells co-treated with a combination of vincristine (20 nM) and Pip2 (2 µM) or vincristine (30 nM) and piperine (2 µM). However, cells treated with lower concentration of vincristine alone (5 nM) were found to be similar to that of the control cells showing green fluorescence with highly organized morphology, suggesting no apoptotic incidence.

## Discussion

Development of multi-drug resistance (MDR) in cancer is a major concern during chemotherapy. P-glycoprotein (P-gp) is one of the ABC proteins that acts as a transporter to efflux a number of chemotherapeutic drugs out of the cancer cells, reducing their efficacy, and plays a major role in the development of MDR. A number of P-gp inhibitors have been tested in the clinical trials, but their use has been limited for toxicity reasons. Nevertheless, there is ongoing interest in the targeting of P-gp to overcome drug resistance and many scientists have reported natural as well as synthetic small molecule compounds and other strategies (siRNA) that can reverse P-gp mediated chemoresistance^49–55^.

Most of the P-gp inhibitors tested in clinical trials have logP > 5 and molecular weight > 500 Da. These two molecular properties are associated with drug-likeness, but much lower values of both (logP < 5; molecular weight < 500 Da) are considered optimal for drug-like molecules. The unfavorable molecular properties of the P-gp inhibitors tested could be a reason for their failure. Accordingly, novel P-gp inhibitors of lower molecular weight and lipophilicity are urgently needed.

Piperine is a low molecular weight natural compound reported to have P-gp inhibitory activity^38^. Herein, we sought to chemically modify piperine so as to enhance its P-gp inhibition, and develop lead molecules. A recent structure-activity relationship analysis of natural products for P-gp inhibition showed the importance of methoxy group for better activity, and several synthetic inhibitors of P-gp were also found to bear methoxy groups^34, 50, 56^. Therefore, the piperidine ring of piperine was replaced with a 6,7-dimethoxytetrahydroisoquinoline moiety, which is also present in the third generation inhibitors tariquidar and elacridar, to give Pip1 (ml.wt: 393.43; logP: 3.38). Another analog, Pip2 (ml.wt: 381.42; logP: 3.40), was designed by replacing the piperidine ring with a 2-(3,4-dimethoxyphenyl)ethylamine moiety; this is also present in the first generation P-gp inhibitor verapamil.

Pip1 and Pip2, were tested *in silico* for their ability to interact with P-gp. In silico studies have shown that piperine is able to interact both at the drug binding site in the transmembrane region as well as in the nucleotide binding domain (NBD) of P-gp, which is responsible for the P-gp inhibition^57, 58^. Here, we used the drug binding site in the transmembrane region for the docking studies. As the crystal structure of human P-gp (hP-gp) is not available, the structure was modeled. The model was based on the *C. elegans* P-gp crystal structure (46% similarity to hP-gp), and not the mouse P-gp crystal structure (87% similarity to hP-gp), as the mouse P-gp structures had errors^59^. Moreover, the hP-gp structure modeled based on *C. elegans* P-gp structure was found to be more compatible with the biochemical studies than the model based on the mouse P-gp structure^60^. Using the hP-gp model, Pip1 and Pip2 were found to interact in the drug binding region, mainly by forming hydrophobic interactions with the residues Met69, Phe72, Phe336, Leu339, Phe728, Tyr953, Phe957, Phe978, Val982 and Met986. Since hydrophobic interactions between P-gp and the drug are thought to be the major interactions responsible for the P-gp inhibition effect^61^, this finding suggests that Pip1 and Pip2 should be able to inhibit P-gp. Based on these results, both Pip1 and Pip2 were synthesized and tested for their potential to inhibit P-gp and reverse resistance in a cancer cell line.

The major factors responsible for chemotherapeutic resistance are: reduced drug accumulation, increased drug export, alterations in drug targets and signal transduction molecules, increased repair of drug-induced DNA damage, and evasion of apoptosis^62^. KB Ch^R^ 8–5 is a drug resistant cancer cell line which is reported to have high levels of P-gp expression^63^, and SW480-VCR cells were obtained by continuous exposure to VCR and observed to have high P-gp expression. These two resistant cell lines were selected to study the P-gp modulator activity and reversal of resistance by piperine analogs. Consistent with previous reports^44, 64^, a decrease in the sensitivity of KB Ch^R^ 8–5 cells towards VCR, COL, and PTX was observed, as compared to the parental sensitive KB cell line. Similarly, a decrease in the sensitivity of SW480-VCR cells towards VCR and PTX was observed, as compared to the parental SW480 cells. Further, western blot and immunofluorescence analysis confirmed that the KB Ch^R^ 8–5 and SW480-VCR cells overexpress P-gp, and that it was localized to the plasma membrane. The accumulation of Rho123, a fluorescent substrate of P-gp^47^, inside the cells depends on the level of P-gp protein. Increased fluorescence intensity of Rho123 in the parental cell line (KB and SW480) compared to that in the resistant cells (KB Ch^R^ 8–5 and SW480-VCR) suggest high levels of P-gp in resistant cells. These results imply that the drug resistance in KB Ch^R^ 8–5 and SW480-VCR is due to the overexpression of P-gp, which could effectively efflux the chemotherapeutic drugs out of the cell.

**Figure 12:**
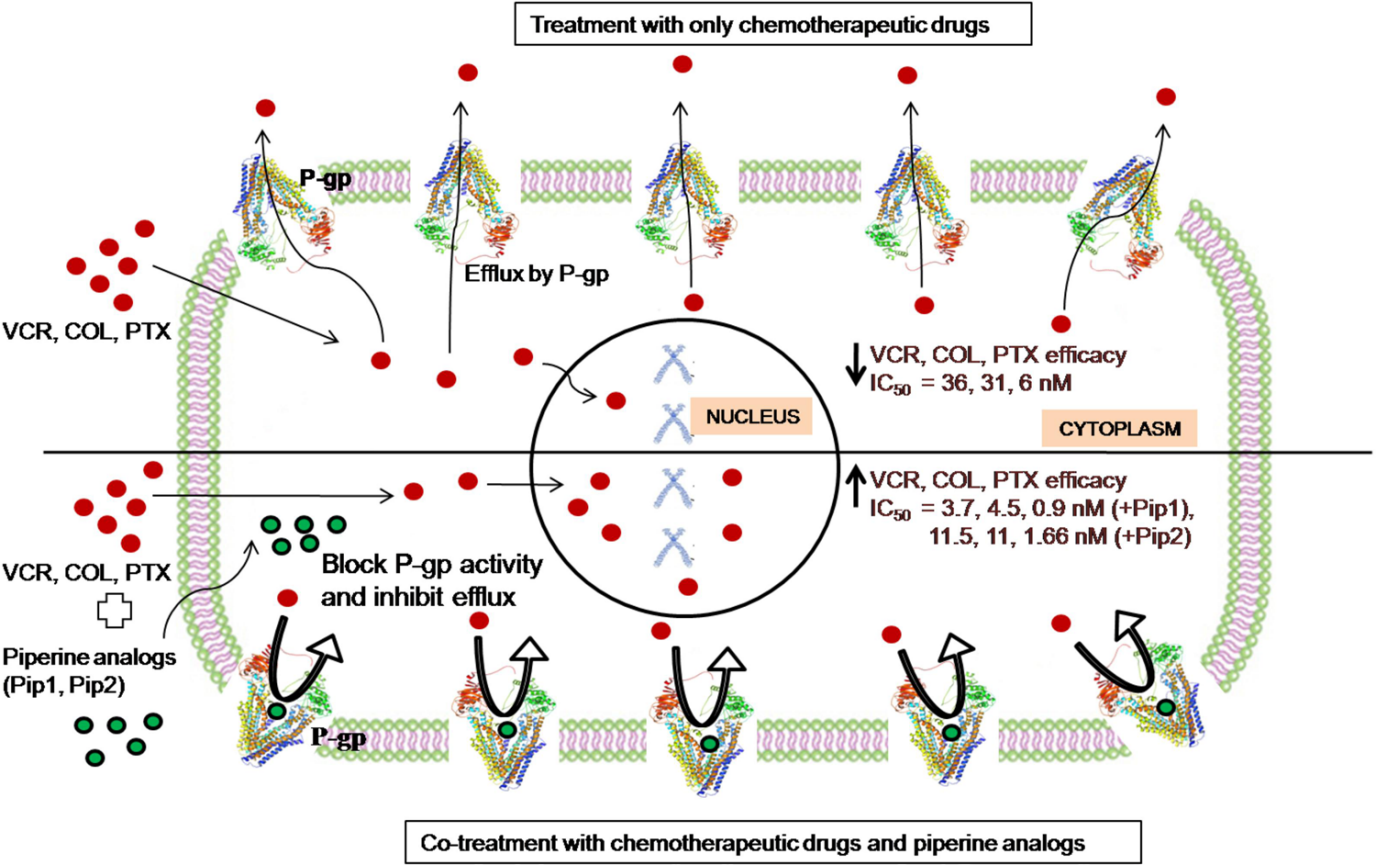
Pictorial representation showing how piperine analogs increased the efficacy of chemotherapeutic drugs in P-gp overexpressing resistant cancer cells.

Co-treatment of VCR/COL/PTX with verapamil or piperine analogs resulted in restoration of VCR/COL/PTX sensitivity in KB Ch^R^ 8–5 and VCR/PTX sensitivity in SW480-VCR cells. This result was further emphasized by the long term clonogenic assay in which co-treatment of VCR with verapamil or piperine analogs significantly reduced the survival of resistant KB Ch^R^ 8–5 cells compared to the cells treated with VCR alone. Furthermore, an increase in the apoptotic bodies in cells co-treated with VCR and piperine analogs compared to the cells treated with VCR alone suggests that the combination treatment is much more effective in the resistant KB Ch^R^ 8–5 cells. Moreover, piperine analogs increased Rho123 accumulation inside the resistant KB Ch^R^ 8–5 and SW480-VCR cells compared to the cells incubated with Rho123 alone; this suggests that the piperine analogs inhibit the P-gp efflux activity and cause Rho123 accumulation. Taken together, the increased sensitivity of KB Ch^R^ 8–5 and SW480-VCR cells to VCR, COL, and PTX on co-treatment with piperine analogs suggests that the piperine analogs increased the concentration of these chemotherapeutic drugs inside the resistant cells by inhibiting the efflux activity of P-gp. The reversal of drug resistance in KB Ch^R^ 8–5 and SW480-VCR cells by piperine analogs, particularly Pip1, was superior to that of piperine – thereby suggesting it as a suitable lead candidate for further studies. Importantly, both compounds were found to be non toxic in non-cancer human embryonic kidney cells (HEK 293: 100% viability at 4 µM; supplementary result Fig. S15) at the concentration at which they exerted P-gp inhibitory activity in drug resistant cancer cells. The overall mechanism of how piperine analogs increased the efficacy of chemotherapeutic drugs in P-gp overexpressing resistant cancer cells is shown in Fig. 12.

In summary, natural products are a rich resource for drug discovery projects in the form of structural leads. A recent analysis showed that over 50% of the new drugs approved between 1981 and 2010 are natural products or nature inspired^65, 66^. Since the number of new chemical entity (NCE) that are approved every year is on the decline, it is essential to mine natural products with more vigour for new drug leads. In this context, two piperine analogs have been designed, synthesized and evaluated for their ability to reverse multi-drug resistance in cancer. The results show that Pip1 ((2*E*,4*E*)-5-(benzo[*d*][1,3]dioxol-5-yl)-1-(6,7-dimethoxy-3,4-dihydroisoquinolin-2(1 *H*)-yl)penta-2,4-dien-1-one) can effectively reverse drug resistance in two cancer cells (KB and SW480) when co-administered with chemotherapeutic agents (vincristine, colchicine and paclitaxel). Further studies (e.g., co-treatment with other chemotherapeutic drugs, testing in other resistant cancer cell lines, *in vivo* activity and toxicity profiling) could help to realize the full potential of these compounds for cancer treatment.

## Materials and Methods

### *In silico* studies

#### Homology modeling

As the tertiary structure of the target human P-gp (hP-gp) protein was not available in protein databank, the 3D structure was modeled using a homology modeling approach. The amino acid sequences of hP-gp were retrieved from UniProt (UniProtKB accession no: P08183) (http://www.uniprot.org) and BLASTp alignment was done to find the best template protein to model hP-gp protein. Mouse P-gp showed 87% identity with that of hP-gp, but registry errors in the mouse P-gp structure have been reported which could impact the *in silico* studies^34, 67^. Another possibility for modeling hP-gp was *Caenorhabditis elegans* (*C. elegans*) P-gp (PDB ID: 4F4C) having a resolution of 3.4 Å; though it shares less sequence identity (46%) to the hP-gp, the similarities in amino acid sequence between these two proteins makes *C. elegans* P-gp a reliable template to model the hP-gp. Sequence alignment of the *C. elegans* P-gp protein and hP-gp protein was performed using ClustalW (https://www.ebi.ac.uk/Tools/msa/clustalw2/). The aligned sequences were used to generate hP-gp models using modeller 9v12 and the best model was selected based on the discrete optimized protein energy (DOPE) and molecular PDF (molpdf score), and validated using PROCHECK and SAVES server (http://services.mbi.ucla.edu/PROCHECK/) by generating a Ramachandran plot. The selected hP-gp model was used further for molecular docking and molecular dynamics simulation studies.

#### Molecular docking

For the docking, the chemical structure of the ligands were drawn using Marvin Sketch v5.6.2 (https://www.chemaxon.com/products/marvin/marvinsketch/) and the 3D structures were saved in molecule object toolkit (.mol) file format. The ligands were prepared using the “LigPrep” wizard of Maestro v9.2 with an Optimized Potentials for Liquid Simulations (OPLS) 2005 force field at pH 7.0 ± 0.4, and allowed to generate possible tautomers and stereo isomers. The modeled protein was prepared by assigning or correcting bond orders and adding hydrogen atoms using the “protein preparation wizard” in the Prime module of Maestro v9.2. Later, the OPLS 2005 force field was used to optimize and minimize the protein at neutral pH.

Molecular docking studies of the piperine analogs with the modeled hP-gp was carried out using Glide extra precision (XP) implemented in Maestro v9.2^68^. The grid was generated using the receptor grid generation panel by denoting the experimentally reported drug binding residues located mainly in the transmembrane (TM) helices TM4, TM5, TM6, TM9, TM10, TM11 and TM12; Leu65, Met69, Phe72 (TM1), Thr199 (TM3), Ser222 (TM4), Ile306, Tyr307 (TM5), Phe336, Leu339, Ile340, Ala342, Phe343 (TM6), Phe728 (TM7), Ala841 (TM9), Ile868, Gly872 (TM10), Phe942, Thr945, Tyr953, Phe957 (TM11), Leu975, Phe978, Val981, Val982, Phe983, Gly984, Ala985 and Met986 (TM12)^67, 69–72^. Under the advanced setting option, a total of 800 poses per ligand for energy minimization and maximum number of minimization steps were set to 100. All other parameters were set to the default values. The top ranked conformation pose of the ligands were selected for further investigation. The binding free energy (ΔG) of the ligands were calculated using Prime MM/GBSA method.

#### Molecular dynamics simulation

Molecular dynamics (MD) simulations were performed using the GROningenMAchine for Chemical Simulations (GROMACS v5.0.4) package^73^. For this purpose, the modeled protein/protein-ligand complex was embedded in a homogeneous 1-palmitoyl-2-oleoylphosphatidylcholine (POPC) membrane bilayer and subjected to MD simulation. Initially, orientation of the modeled protein with respect to the membrane was generated by using Positioning of Proteins in Membrane (PPM) server (http://opm.phar.umich.edu/server.php), which calculates rotational and translational positions of transmembrane and peripheral proteins in membranes using their 3D structure^74^. Later, the generated atomic coordinates of the modeled hP-gp with the lipid bilayer boundaries (indicated by dummy atoms; supplementary result Fig. S3) was used to generate the protein-membrane complex on CHARM-GUI membrane builder server (http://www.charmm-gui.org/)^43, 75, 76^. The system was generated by defining the following parameters: a rectangular box shaped system in the homogeneous POPC bilayer containing the P-gp was selected with 1.5 lipid bilayers around the protein (1.5 lipid bilayer corresponds to 3 molecules of POPC). Further, water layers of 20 Å thickness above and below the lipid bilayer was added to fully solvate the protein and the system was neutralized by adding 0.15 M KCl ions. Finally, the generated output files from CHARMM-GUI were used for the next MD simulation step using GROMACS. Initially, the energy of the system was minimized using steepest descent method, followed by equilibration at room temperature of 300 K and 1 bar pressure, with the Berendsen weak coupling method to regulate the temperature and pressure of the system^77^. Finally, MD simulation production run was carried out for 50 ns to check the stability of the protein alone and in complex with the ligands.

### Synthesis

#### General methods

Chemicals and solvents used for the synthesis are of reagent grade and were used without further purification. Reactions were carried out under dry nitrogen atmosphere and monitored by TLC using Merck 60 F_254_ silica gel glass backed plates (5 cm × 10 cm); zones were detected visually under UV irradiation (254 nm). A Varian Mercury-400 spectrometer was used to record ^1^H NMR spectra and an Agilent MSD-1100 ESI-MS/MS system was used to record low-resolution mass spectra (LRMS). Purity of the synthesized compounds was determined with an Hitachi 2000 series HPLC system using C-18 column (Agilent ZORBAX Eclipse XDB-C18 5 μm, 4.6 mm × 150 mm)^78^.

#### General procedure for the synthesis of Pip1 and Pip2

N-methylmorpholine (0.24 mmol) was added to a stirred solution of piperic acid (0.12 mmol) in tetrahydrofuran (THF) (2 mL) at 0 °C. Isobutyl chloroformate (0.12 mmol) was added dropwise with stirring, over 10 minutes. Appropriate amine (0.24 mmol) was added to the reaction mixture and after the completion of the reaction, water was added and extracted with ethyl acetate. The organic layer was washed with brine, 10% NaHSO_4_ and saturated NaHCO_3_; dried over anhydrous Na_2_SO_4_, and concentrated in vacuo. The residue was then purified by silica gel (Merck Kieselgel 60, no. 9385, 230−400 mesh ASTM) column chromatography using an appropriate mobile phase (see below) to give the piperine analogs Pip1 and Pip2.

#### (2*E*,4*E*)-5-(benzo[*d*][1,3]dioxol-5-yl)-1-(6,7-dimethoxy-3,4-dihydroisoquinolin-2(1 *H*)-yl)penta-2,4-dien-1-one (Pip1)

6,7-Dimethoxy-1,2,3,4-tetrahydro-isoquinoline was used as the amine and column chromatography was carried out using a mixture of hexane/ethyl acetate = 2/1 to give **Pip1** (Yield, 25%). ^1^H NMR (400 MHz, CDCl_3_):*δ *2.85 (bs, 2 H), 3.79 (bs, 2 H), 3.86 (s, 6 H), 4.72 (bd, 2 H), 6.01 (s, 2 H), 6.49 (d, *J* = 14.4 Hz, 1 H), 6.80–6.64 (m, 5 H), 6.90 (dd, *J* = 8.2, 1.2, Hz, 1 H), 6.99 (d, *J* = 1.2 Hz, 1 H), 7.46 (dd, *J* = 11.2, 8.0 Hz, 1 H); LCMS (ESI) m/z: 394.1 [M + H]^+^. HPLC purity: 97%.

#### (2*E*,4*E*)-5-(benzo[*d*][1,3]dioxol-5-yl)-*N*-(3,4-dimethoxyphenethyl)penta-2,4-dien-amide (Pip2)

2-(3,4-Dimethoxy-phenyl)-ethylamine was used as the amine and column chromatography was carried out using a mixture of hexane/ethyl acetate = 2/1 to give **Pip2** (Yield, 51%). ^1^H NMR (400 MHz, CDCl_3_): *δ* 2.82 (t, *J* = 6.8 Hz, 2 H), 3.60 (q, *J* = 6.4 Hz, 2 H), 3.87 (s, 6 H), 5.48 (bs, 1 H), 5.84 (d, *J* = 14.8 Hz, 1 H), 5.98 (s, 2 H), 6.65 (dd, *J* = 15.6, 10.8 Hz, 1 H), 6.83–6.74 (m, 5 H), 6.89 (d, *J* = 8 Hz, 1 H), 6.97 (s, 1 H), 7.53 (dd, *J* = 14.8, 10.8 Hz, 1 H). LCMS (ESI) m/z: 382.1 [M + H]^+^. HPLC purity: 98%.

### *In vitro* studies

#### Materials

Verapamil hydrochloride, piperine, vincristine sulfate, rhodamine 123 (Rho123), dimethyl sulfoxide (DMSO), RIPA buffer, and protease inhibitor cocktail were purchased from Sigma-Aldrich, USA. Colchicine, Dulbecco’s Modified Eagle Medium (DMEM), 3-(4,5-dimethylthiazol-2-yl)-2,5-diphenyltetrazolium bromide (MTT), trypsin-ethylene diamine tetra acetic acid (Trypsin–EDTA) solution, penicillin–streptomycin solution, phosphate-buffered saline (PBS), acridine orange (AO), ethidium bromide (EB), 4′,6-Diamidino-2-Phenylindole, dihydrochloride (DAPI), Bradford reagent and fetal bovine serum (FBS) were obtained from HiMedia (Mumbai, India). Nitrocellulose membrane was purchased from GE healthcare, USA. Anti-P-gp (D-11), and anti-tubulin antibodies were purchased from Santa Cruz Biotechnology (Santa Cruz, CA, USA).

#### Cell culture

Human KB 3–1, KB Ch^R^ 8–5, SW480 and HEK 293 cells were obtained from the National Centre for Cell Science (NCCS), Pune, India. The cells were grown in Dulbecco’s modified Eagle’s medium supplemented with 10% FBS, penicillin (100 U/ml), streptomycin (100 mg/ml), 1% L-glutamine and 1% non-essential amino acids at 37 °C in a humidified atmosphere and 5% CO_2_. The resistant KB Ch^R ^8–5 cells were derived from the parental KB cells and were continuously grown in culture media containing 10 nM of colchicine in order to maintain the resistance. The resistant SW480-VCR cells were obtained by continuous exposure of parental SW480 cells to vincristine from a starting concentration of 0.625 nM. When the cells were able to grow at a given concentration of vincristine, they were harvested and plated in a new flask and exposed to a 2-fold higher concentration of vincristine. Likewise, the cells were exposed to higher concentration of vincristine and after 3 months of continuous exposure, the final obtained resistant SW480-VCR cells could able to grow in the presence of 20 nM of vincristine. The cells were cultured in tissue culture flasks. When they reached confluence, they were harvested and plated in new flasks at 1:2 ratios. Up to 10–15 passages were used for drug treatment.

#### Cell viability assay

The cell viability was evaluated using MTT. Briefly, cells were seeded at a density of 5 × 10^3^ cells/well in a 96-well plate and incubated for 24 h to retain their morphology. The next day, cells were incubated with vincristine/colchicines/paclitaxel alone or in combination with verapamil, piperine, Pip1 or Pip2 for 72 h at 37 °C in a humidified atmosphere and 5% CO_2_. The final concentration of DMSO was <0.1%. After 72 h incubation, 100 µl of MTT solution (5 mg/ml) was added to each well and the plate was further incubated for 4 h at 37 °C. Subsequently, MTT was removed and the formazen crystals formed were dissolved by adding 100 µl of DMSO. The absorbance was measured using multimode reader (Molecular Devices) at 570 nm. The proportion of cell survival (%) was calculated using the formula (OD of drug treated sample - blank)/(OD of control - blank) × 100%. Fold-resistance (FR) was calculated by dividing the IC_50_ value for vincristine of parental KB 3–1 and vincristine resistant KB Ch^R^ 8–5 cells in the absence or presence of verapamil, piperine, Pip1, Pip2 by IC_50_ value for vincristine of parental KB 3–1 cells. Fold-resistance reversal (FRR) was calculated by dividing the FR value for vincristine alone by FR value for vincristine in the presence of verapamil, piperine, Pip1 or Pip2.

#### Clonogenic assay

Cells were seeded at a density of 10^3^ cells/well in 12-well plate and incubated for 24 h at 37 °C in a humidified atmosphere and 5% CO_2_. After 24 h incubation, media was replaced with fresh media containing different concentrations of vincristine alone (5–40 nM) or along with 2 µM each of verapamil, piperine, Pip1 or Pip2 and incubated for 72 h. The drug medium was replaced with drug free medium and further incubated for 7–10 days. Later, cells were fixed and stained with 1% methylene blue in 50% ethanol. Colonies were observed under the microscope and manually counted.

#### Western blot analysis of P-gp expression

The cells were lysed with ice-cold RIPA buffer containing 150 mM NaCl, 1.0% IGEPAL^®^ CA-630, 0.5% sodium deoxycholate, 0.1% SDS, 50 mM Tris, and protease inhibitors at pH 8.0. The lysate was then centrifuged at 12,000 rpm at 4 °C for 15 min and the supernatant was collected. The protein concentration was determined by Bradford method. 40 µg protein sample was loaded, separated on 10% SDS-PAGE and transferred onto nitrocellulose membrane. Membrane was blocked with 3% BSA for 1 hr at room temperature and incubated with the mouse monoclonal anti-P-gp antibody (1:1000)/mouse monoclonal anti-tubulin antibody (1:1000) for overnight at 4 °C. The membrane was washed five times for five minutes each in TBST. Subsequently, the membrane was incubated with anti-mouse HRP-conjugated secondary antibody (1:10000) for 45 min at room temperature. Later, the membrane was washed five times, five minutes each in TBST and the bands were visualized by treating the membrane with HRP substrate, 3,3′,5,5′-tetramethylbenzidine (TMB)/H_2_O_2_.

#### Immunofluorescence detection of P-gp localization

Cells were seeded onto the cover slips and incubated for 24 h to attach. Cells were fixed and permeabilized with methanol for 10 min at −20 °C. Cells were washed three times, five minutes each in PBS, and then blocked with 1% BSA in PBST for 1 h at room temperature. Later, cells were incubated with mouse monoclonal anti-P-gp antibody diluted 1:100 (Santa Cruz, USA) in 1% BSA in PBST in a humidified chamber for 1 h at room temperature. Subsequently, cells were washed five times, five minutes each in PBS and incubated with anti-mouse IgG secondary antibody conjugated with FITC (1:100) (Santa Cruz, USA), for 1 h at room temperature in the dark. Finally, cells were washed five times, five minutes each in PBS and mounted on a glass slide using 90% glycerol. Images were acquired using a Nikon Eclipse Ti-S inverted Fluorescence microscope (Nikon Instruments, Melville, New York).

#### Rhodamine 123 accumulation assay

Cells were seeded in a 6 well plate and incubated for 24 h at 37 °C in a humidified atmosphere and 5% CO_2_. After 24 h incubation, cells were either incubated for 2 h with media or with media containing 4 µM each of verapamil, piperine, Pip1, or Pip2. Later, Rho 123 (10 µM) was added and further incubated for 1 h at 37 °C in the dark. Cells were washed thoroughly three times with ice cold PBS and images were acquired using Nikon Eclipse Ti-S inverted Fluorescence microscope. Quantification of fluorescence was done using ImageJ software^79^. The corrected total cell fluorescence (CTCF) was calculated using the formula: integrated density−(area of selected cell × mean fluorescence of background readings)^80^.

#### Fluorescence microscopic analysis of cell death

Cell death analysis was carried out by using acridine orange/ethidium bromide (AO/EB) double staining assay as described by Kasibhatla *et al*.,^48^. Briefly, cells were seeded in 6 well plates and incubated for 24 h. Cells were incubated for 24 h without or with vincristine alone (5, 20, 40 nM) or in combination with 2 µM each of verapamil, piperine, Pip1 or Pip2. The drug concentrations were fixed based on the viability and drug sensitivity assay. After being incubated for 24 h, dual stain containing solution AO/EB (100 µg/ml each) was added to each well and images were acquired using a Nikon Eclipse Ti-S inverted fluorescence microscope.

### Statistical analysis

The IC_50_ values were determined from the inhibitor vs response curves in GraphPad Prism 7 (GraphPad Software) using the non-linear regression analysis. For comparison between the groups, one-way analysis of variance (ANOVA) followed by Bonferroni’s *post hoc* test was used. Significant changes are indicated as *p < 0.05, **p < 0.005, ***p < 0.0005 and ****p < 0.0001.

## Electronic supplementary material

Supplementary results

Supplementary video
