# Supplementary material for: Targeting P-glycoprotein: Investigation of piperine analogs for overcoming drug resistance in cancer
Source: Sci Rep. 2017 Aug 11;7:7972. doi: 10.1038/s41598-017-08062-2 (PMC5554262; doi:10.1038/s41598-017-08062-2)
Supplement: Supplementary file 1 — Supplementary results [file 41598_2017_8062_MOESM1_ESM.pdf]

Supplementary results

Targeting P-glycoprotein: Investigation of piperine analogs for overcoming drug resistance in cancer

Safiulla Basha Syed,[a,b] Hemant Arya,[a] I-Hsuan Fu,[c] Teng-Kuang Yeh,[c] Latha Periyasamy,[d] Hsing-Pang Hsieh,[c,e,*] and Mohane Selvaraj Coumar[a,*]

[a]*Centre for Bioinformatics, School of Life Sciences, Pondicherry University, Kalapet, Puducherry- 605014, India.*

[b]*DBT-Interdisciplinary Program in Life Sciences, Pondicherry University, Kalapet, Puducherry- 605014, India*

[c]*Institute of Biotechnology and Pharmaceutical Research, National Health Research Institutes, 35 Keyan Road, Zhunan, Miaoli County 350, Taiwan, ROC.*

[d]*Department of Biochemistry & Molecular Biology, School of Life Sciences, Pondicherry University, Kalapet, Puducherry- 605014, India.*

[e]*Department of Chemistry, National Tsing Hua University, Hsinchu, Taiwan, ROC.*

*Corresponding authors: H.P. Hsieh, email: hphsieh@nhri.org.tw; M.S. Coumar, email: mohane@bicpu.edu.in

Table S1. List of P-gp inhibitors with their molecular weight and calculated logP values (MarvinSketch).

**P-gp inhibitor**	**Ml. wt[a]**	**logP[a]**
Verapamil	454.611	5.04
Cyclosporin 1	1202.635	3.64
Biricodar	603.716	4.77
Laniquidar	586.736	5.62
Elacridar	563.654	6.81
Zosuquidar	527.616	4.82

[a] calculated using MarvinSketch v5.6.2

Table S2. RMSF values (Å) of the binding site residues of P-gp with and without inhibitors during 50 ns MD simulation.

Sl. No	Residue	RMSF (Å)				
		Apo form of P-gp or Protein alone	Verapamil bound P-gp	Piperine bound P-gp	Pip1 bound P-gp	Pip2 bound P-gp
1.	Met69	2.19	1.14	1.02	1.29	1.49
2.	Phe72	1.48	1.38	1.20	1.51	1.51
3.	Tyr307	1.47	1.27	1.13	1.55	0.99
4.	Phe336	1.70	1.12	1.26	1.27	1.37
5.	Leu339	1.33	1.14	1.45	1.45	1.42
6.	Ile340	1.28	1.33	1.11	1.18	1.52
7.	Phe343	1.45	1.28	1.27	1.77	1.68
8.	Phe728	1.65	1.51	1.00	1.72	1.38
9.	Ile868	1.34	1.67	1.60	1.52	1.29
10.	Tyr953	1.51	1.18	1.25	1.29	1.83
11.	Phe957	1.31	1.54	1.44	1.40	1.76
12.	Phe978	1.42	1.33	1.72	1.54	1.82
13.	Val981	1.25	1.06	1.19	1.57	2.21
14.	Val982	1.44	1.19	1.11	1.46	2.14
15.	Phe983	2.10	1.37	1.23	1.87	1.84
16.	Ala985	1.48	1.19	1.00	1.78	1.57
17.	Met986	1.90	1.80	1.68	2.27	1.62

Table S3. Interaction analysis of initial pose (docked) and final pose (after 50ns MD simulation) of ligands in P-gp.

S. No	Compound	Interacting residues (Docked pose)	Interacting residues (After MD simulation)
1	Verapamil	Phe336 (π-π interaction); Met69, Phe72, Phe336, Leu339, Ile340, Phe728, Ile868, Tyr953, Phe957, Phe978, Val981, Val982, Phe983, Ala985 and Met986 (hydrophobic interaction)	Met69, Phe72, Phe336, Leu339, Ile340, Phe343, Phe728, Ile868, Tyr953, Val981, Val982, Phe983, Ala985 and Met986 (hydrophobic interaction)
2	Piperine	Tyr307 (Hydrogen bond); Met69, Phe72, Phe336, Leu339, Phe728, Tyr953, Val982, Phe983 and Met986 (hydrophobic interaction)	Met69, Phe336, Leu339, Ile340, Phe343, Phe728, Phe983 and Met986 (hydrophobic interaction)
3	Pip1	Phe72 and Phe983 (π-π interaction); Met69, Phe336, Leu339, Phe728, Tyr953, Phe978, Val982, Phe983 and Met986 (hydrophobic interaction)	Phe72, Met69, Phe336, Leu339, Ile340, Phe343, Phe728, Ile868, Tyr953, Val982, Phe983, Ala985 and Met986 (hydrophobic interaction)
4	Pip2	Met69, Phe336, Leu339, Phe728, Ile868, Tyr953, Phe957, Phe978, Val981, Val982, Phe983, Ala985 and Met986 (hydrophobic interaction)	Met69, Tyr307, Leu339, Ile340, Phe343, Phe728, Tyr953, Val982 and Phe983 (hydrophobic interaction)

Note: Residues in black color- interactions were maintained in docked and after 50 ns MD simulation
Residues in blue color- Interactions were lost during MD simulation
Residues in red color- Interactions were not seen in docked but formed during MD simulation

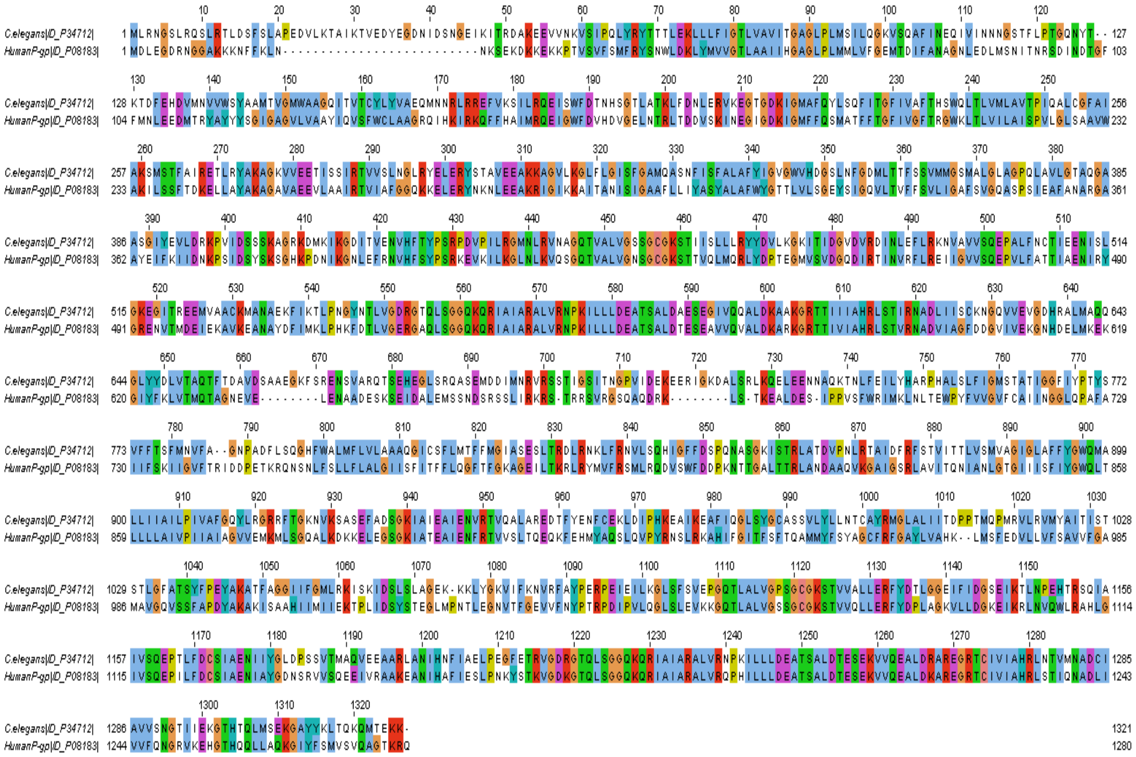

Figure S1: Sequence alignment of *C. elegans* (PDB ID: 4F4C) and human P-gp used to generate the 3D models of P-gp.

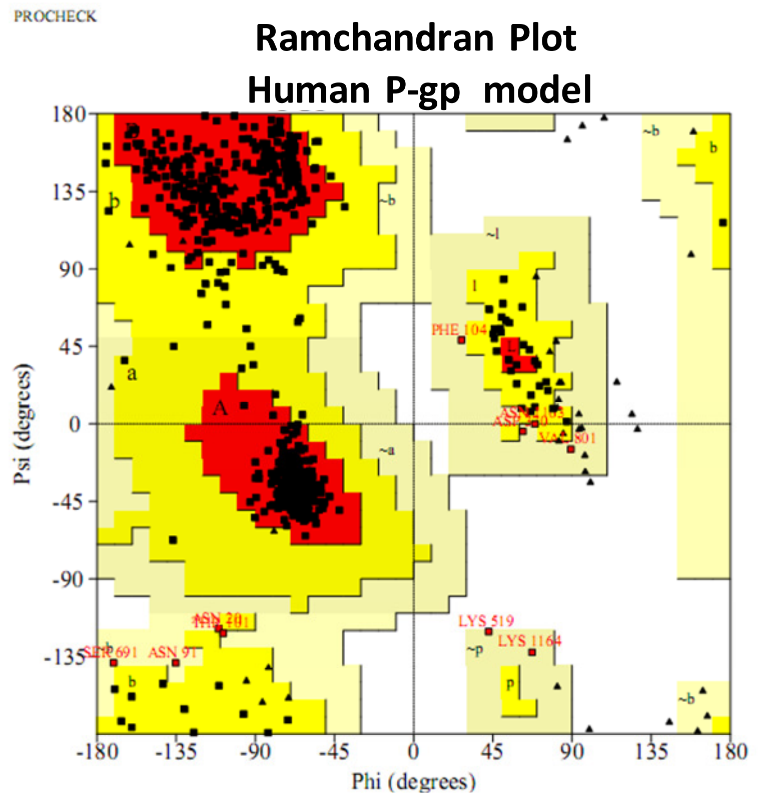

Plot statistics

Residues in most favoured regions [A,B,L]	1064	92.5%
Residues in additional allowed regions [a,b,l,p]	76	6.6%
Residues in generously allowed regions [~a,~b,~l,~p]	10	0.9%
Residues in disallowed regions	0	0.0%
	----	------
Number of non-glycine and non-proline residues	1150	100.0%
Number of end-residues (excl. Gly and Pro)	2	
Number of glycine residues (shown as triangles)	99	
Number of proline residues	29	

Total number of residues	1280	

Based on an analysis of 118 structures of resolution of at least 2.0 Angstroms and R-factor no greater than 20%, a good quality model would be expected to have over 90% in the most favoured regions.

Figure S2: Ramachandran plot of human P-gp homology model

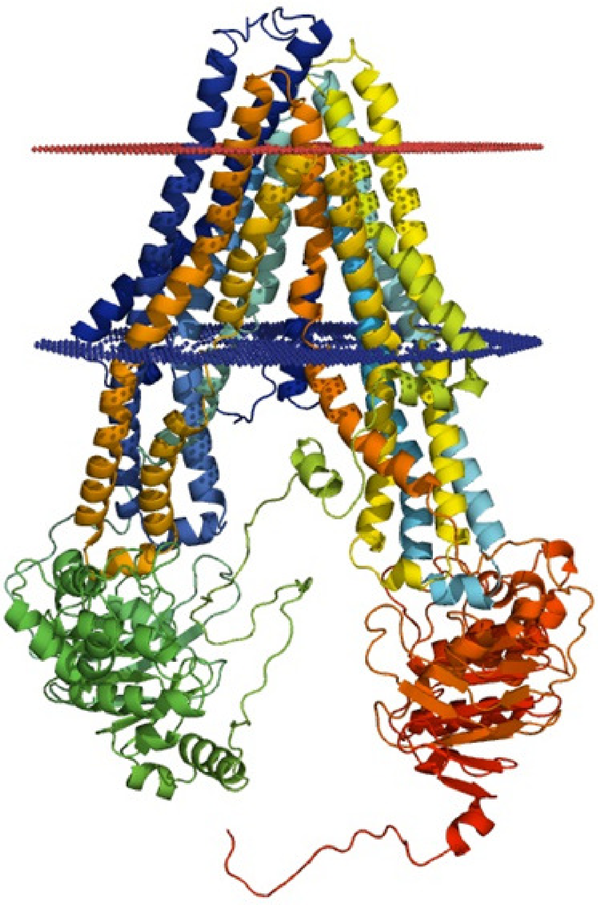

Figure S3: The modelled P-gp protein with the lipid bilayer boundaries which are indicated by dummy atoms generated from the PPM server.

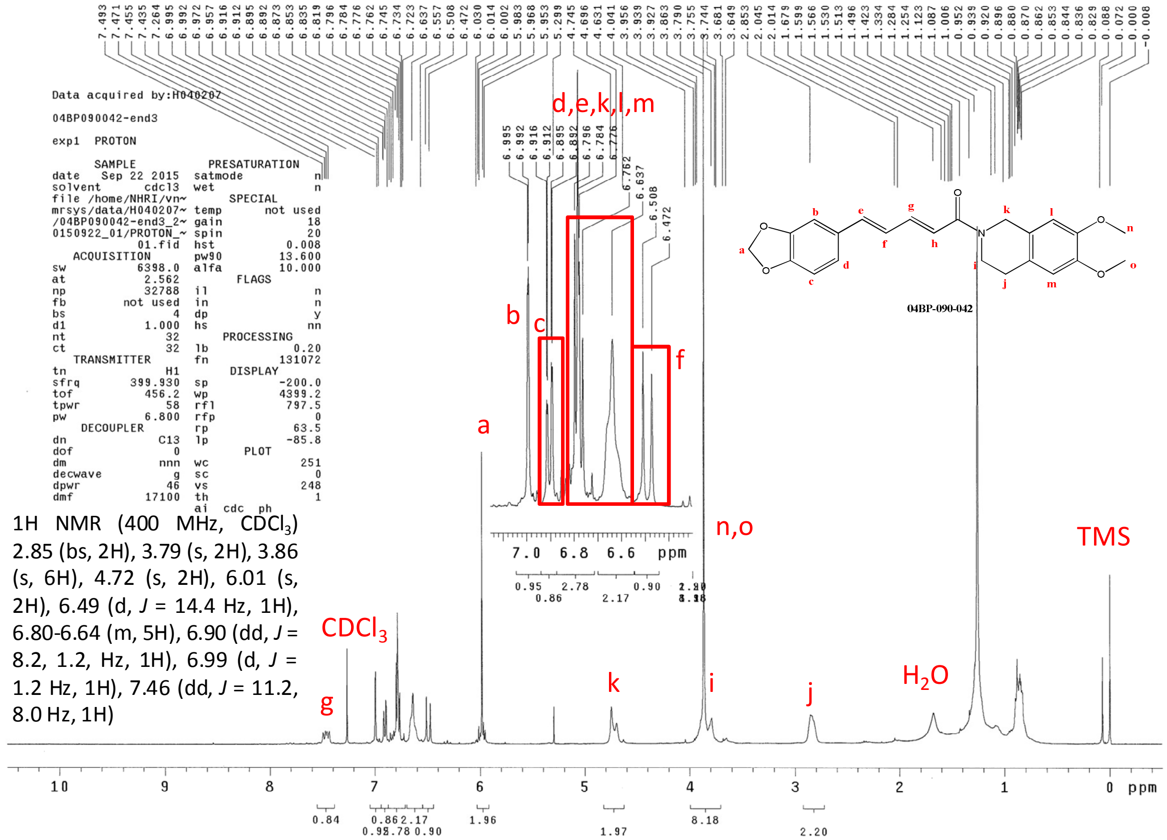

Figure S4: ^{1}H-NMR spectrum of (2E,4E)-5-5(2H-1,3-benzodioxol-5-yl)-1-(6,7-dimethoxy-1,2,3,4-tetrahydroisoquinolin-2-yl)penta-2,4-dien-1-one (**Pip1**) .

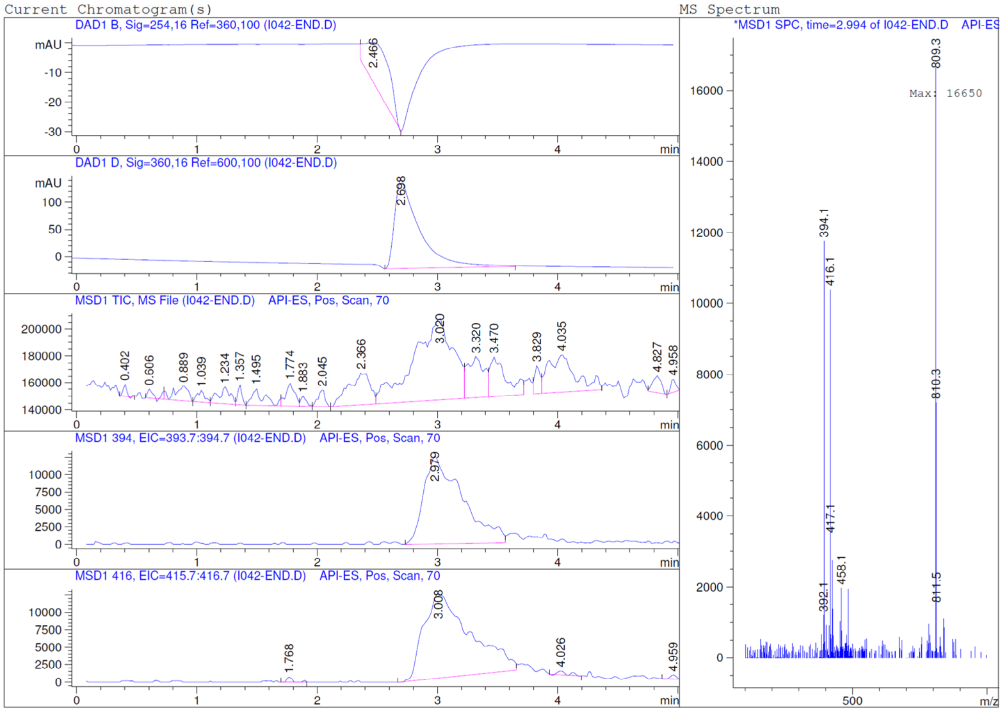

Figure S5: LC-MS analysis of **Pip1**.

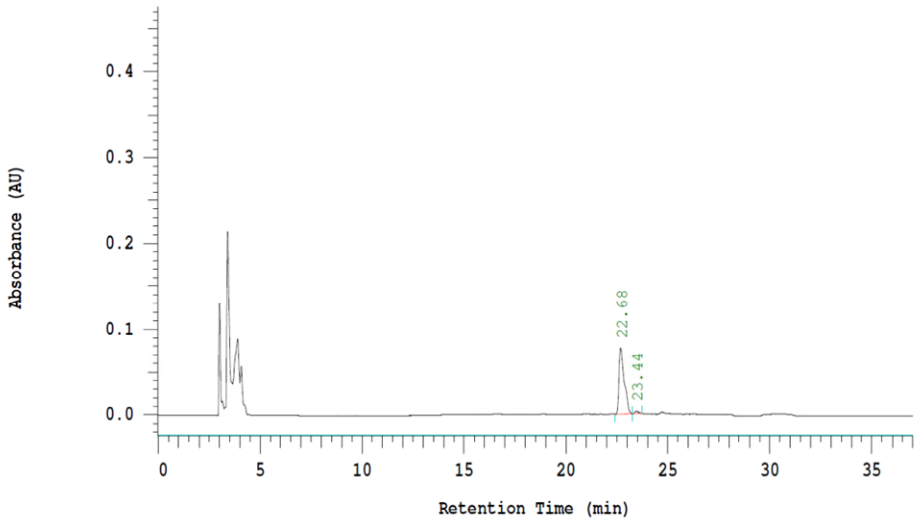

Processing Method: Purity 2007/12/24 37min
Column Type: Column Method Developer: Bob
Method Description:

Peak Quantitation: AREA
Calculation Method: AREA%

No.	RT	Area	Height	Conc 1
1	22.68	657984	38496	97.649
2	23.44	15842	1287	2.351
		673826	39783	100.000

Figure S6: HPLC purity analysis of **Pip1**.

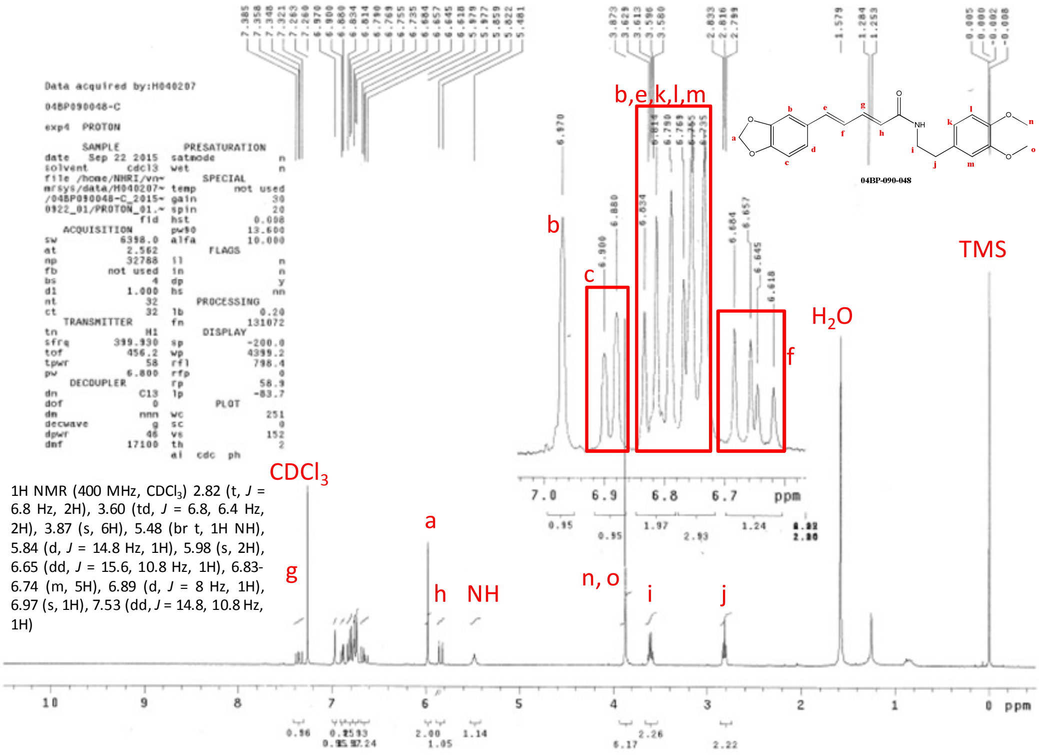

Figure S7: ^{1}H-NMR spectrum of (2E,4E)-5-(2H-1,3-benzodioxol-5-yl)-N-[2-(3,4-dimethoxyphenyl)ethyl]penta-2,4-dien-amide **(Pip2).**

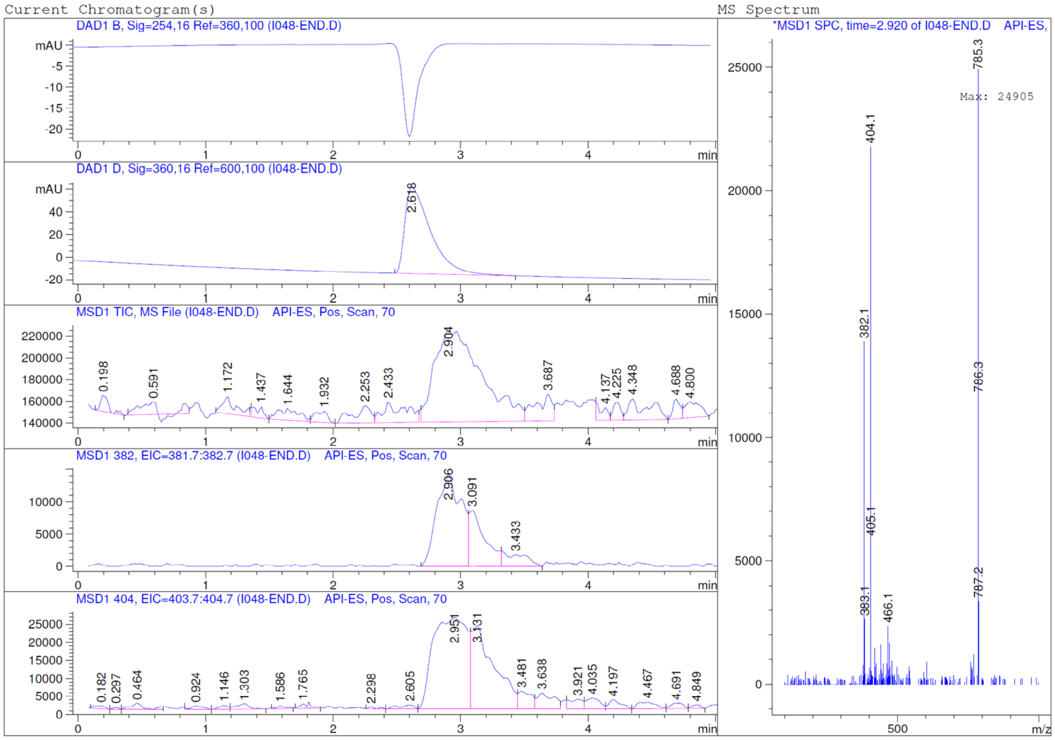

Figure S8: LC-MS analysis of **Pip2**.

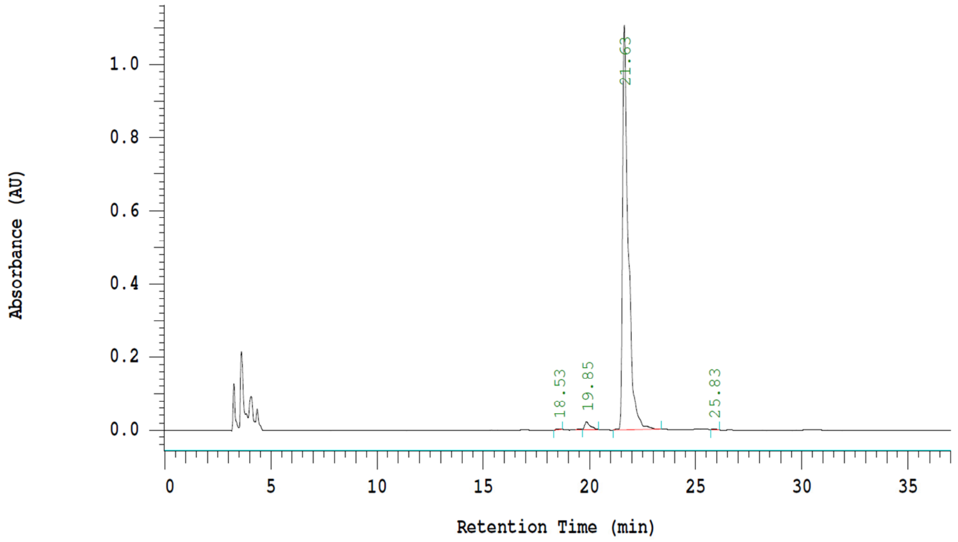

Processing Method: Purity 2007/12/24 37min
Column Type: Column Method Developer: Bob
Method Description:

Peak Quantitation: AREA
Calculation Method: AREA%

No.	RT	Area	Height	Conc 1
1	18.53	11831	993	0.112
2	19.85	181827	10724	1.729
3	21.63	10310175	552923	98.035
4	25.83	12970	1031	0.123
		10516803	565671	100.000

Figure S9: HPLC purity analysis of **Pip2**.

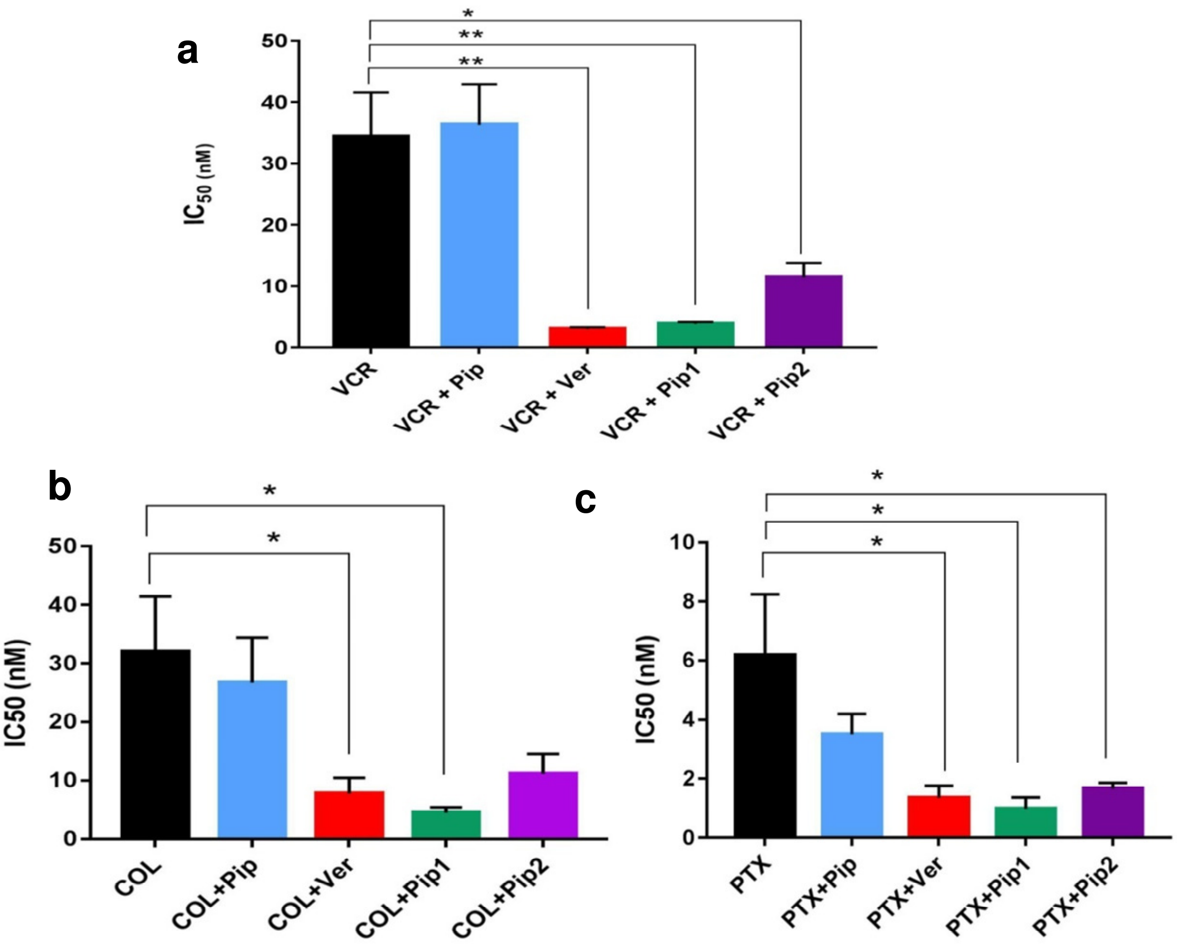

Figure S10: IC_{50} value of (a) vincristine (VCR), (b) colchicine (COL) and (c) paclitaxel (PTX) alone and in combination with 2 μM each of piperine (Pip), verapamil (Ver), Pip1 or Pip2 in KB Ch^R 8-5 cell lines. Error bars represent the mean ± SEM of two or three independent experiments, each done in triplicates. * $p< 0.05$, ** $p< 0.005$ versus control (i.e. VCR/COL/PTX alone).

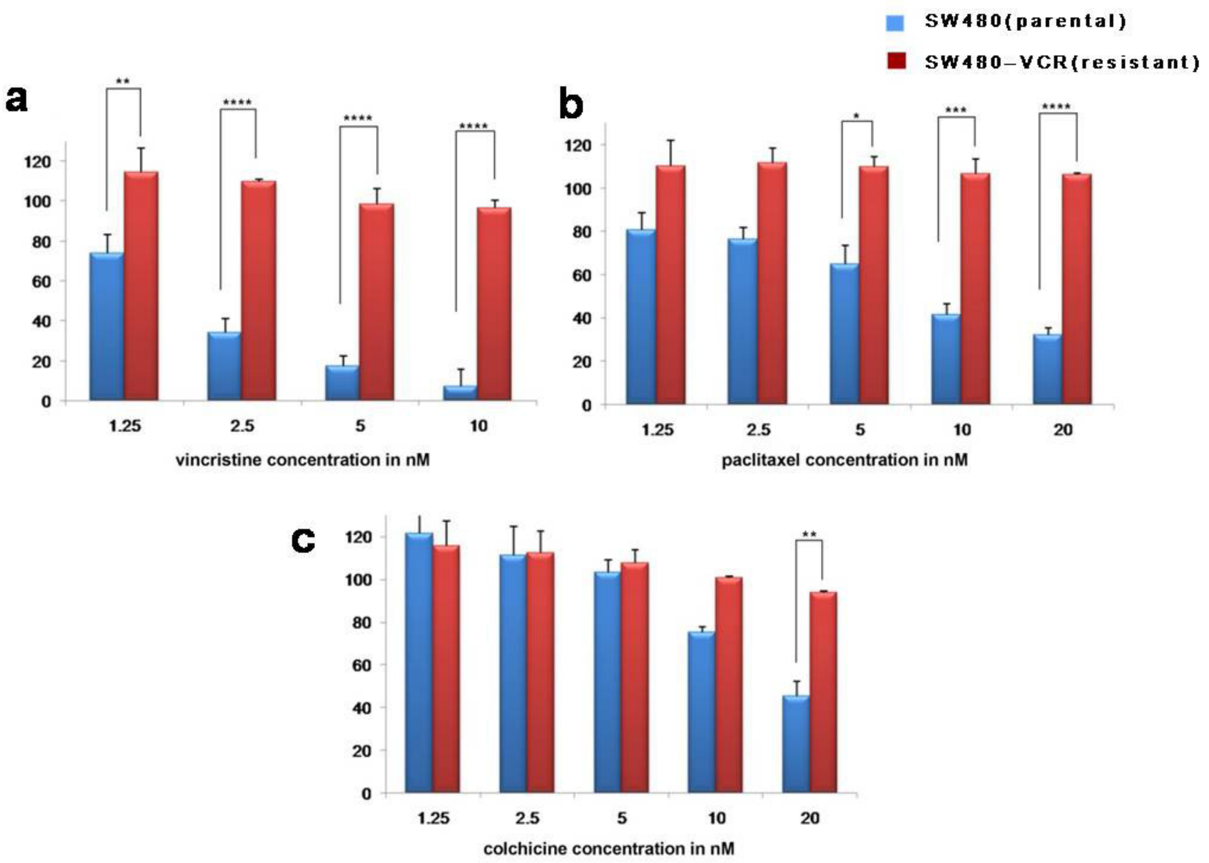

Figure S11: Percentage cell viability of SW480 (parental) and SW480-VCR (resistant) cells. (a) cells were treated with vincristine, (b) cells were treated with paclitaxel, and (c) cells were treated with colchicine. Error bars represent standard error of mean (SEM) of two independent experiments, each done in triplicates. * $p < 0.05$, ** $p < 0.005$, *** $p < 0.0005$ and **** $p < 0.0001$.

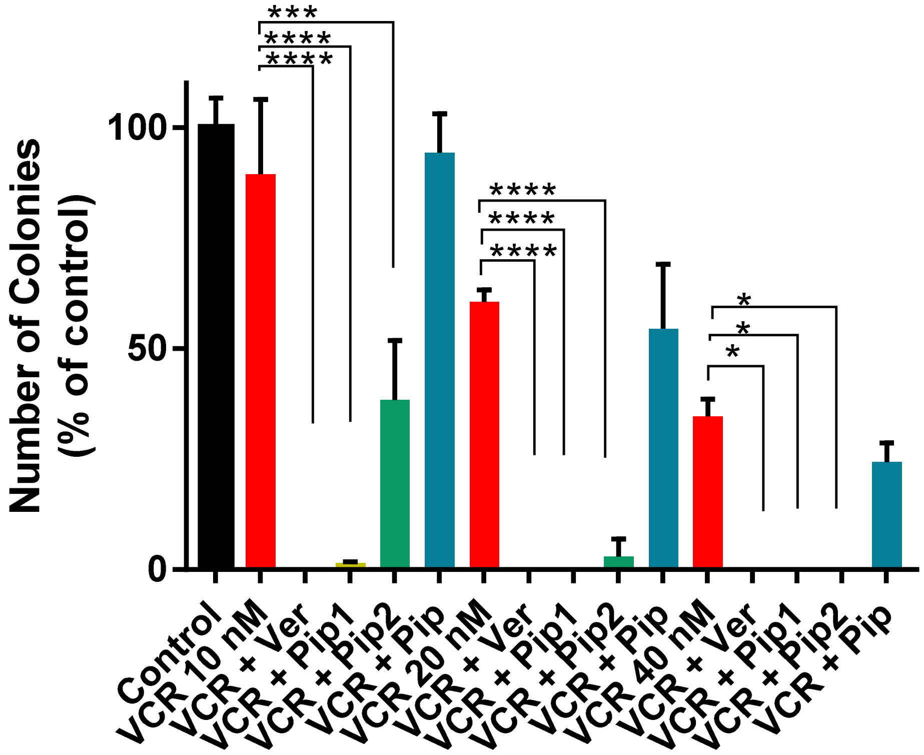

Figure S12: Colony forming ability of resistant KB Ch^R 8-5 cells. The cells were treated with vincristine (VCR; 10-40 nM) alone or in combination with 2 µM each of verapamil (Ver), piperine (Pip), Pip1, and Pip2 for 72 h. Cells incubated only with culture media served as a control. The colonies were quantified as a percentage of control treatment. Error bars represent standard error of mean (SEM) of three independent experiments. * $p< 0.05$, *** $p< 0.0005$ and **** $p < 0.0001$ versus VCR 10 nM/20 nM/40nM alone.

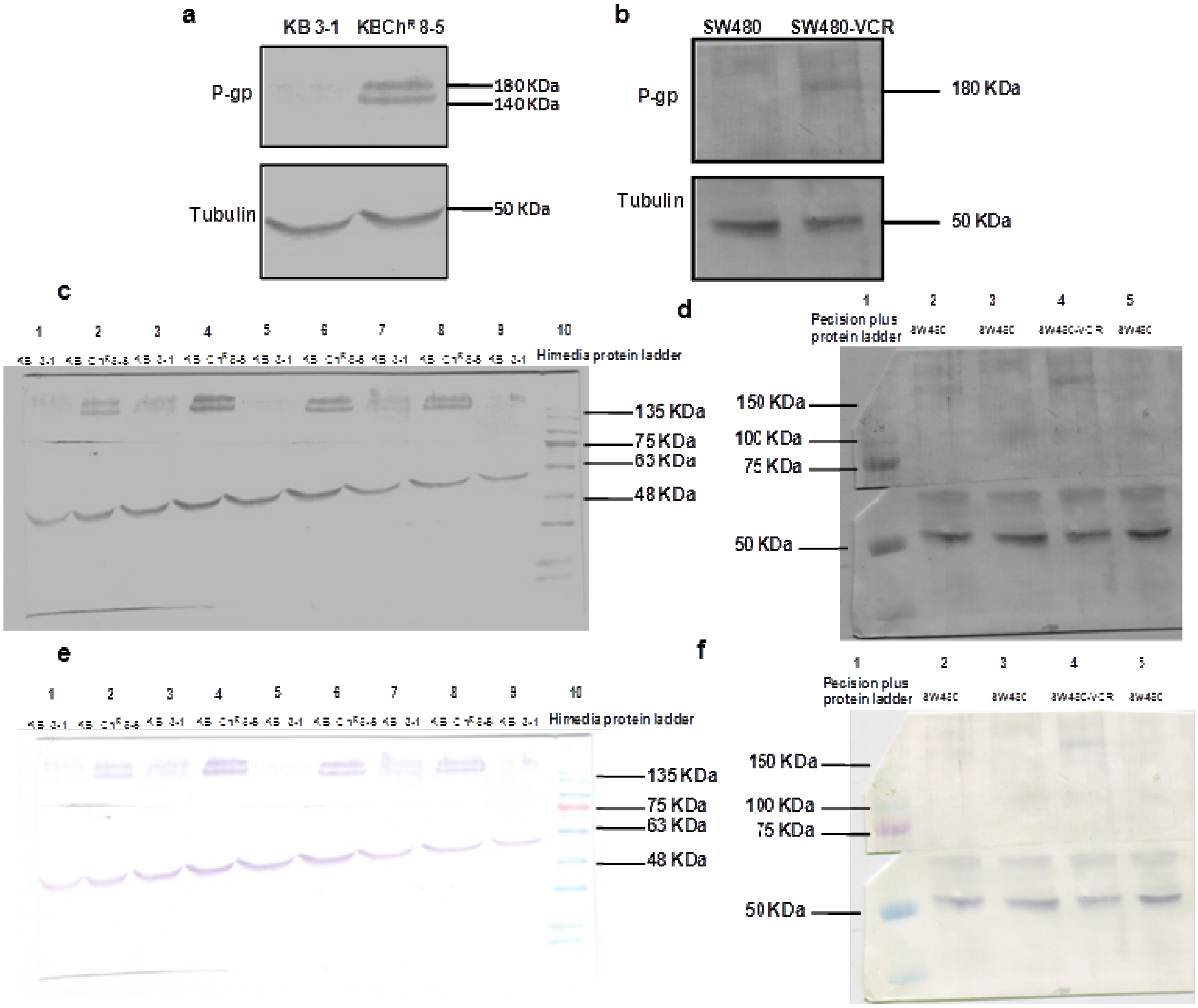

Figure S13: Western blot analysis of P-gp protein in (a) KB 3-1 (parental) and KB Ch^{R}8-5 (resistant) cells and (b) SW480 (parental) and SW480-VCR (resistant) cells. The double band of P-gp in KB Ch^R 8-5 cells reflects the glycosylation status of the protein. The upper 180 kDa band corresponds with the mature/fully glycosylated P-gp, whereas the lower 140 kDa band represents the immature/partially glycosylated P-gp [1].

Gray scale of the original blots of (c) KB parental (KB 3-1), resistant (KB Ch^R 8-5) and (d) SW480 parental, resistant (SW480-VCR). Original scanned blots of (e) KB parental (KB 3-1), resistant (KB Ch^R 8-5) and (f) SW480 parental, resistant (SW480-VCR).

*Note: Two different gels were run and the proteins were transferred onto two different membranes. (c) Gray scale of the membrane having KB 3-1 and KB Ch^R 8-5 proteins and (d) Gray scale of the membrane having SW480 and SW480-VCR proteins. After the protein was transferred onto the nitrocellulose membrane, the membrane was cut based on the molecular weight of the protein ladder (Himedia-MBT092 or precision plus protein-1610374) and incubated with the corresponding primary antibodies. Later, the membrane was incubated with the HRP-conjugated secondary antibody. Finally, the bands were visualized with HRP substrate, 3,3',5,5'-tetramethylbenzidine (TMB)/H_2O_2. The membranes were scanned and saved as an image (e and f). The images were converted to gray scale and cropped to show the desired proteins, P-gp and tubulin of both, KB parental and resistant (Lane 5 and Lane 6) are displayed in Fig. S13 (a) and P-gp and tubulin of both SW480 parental and resistant (Lane 3 and Lane 4) are displayed in Fig. S13 (b).

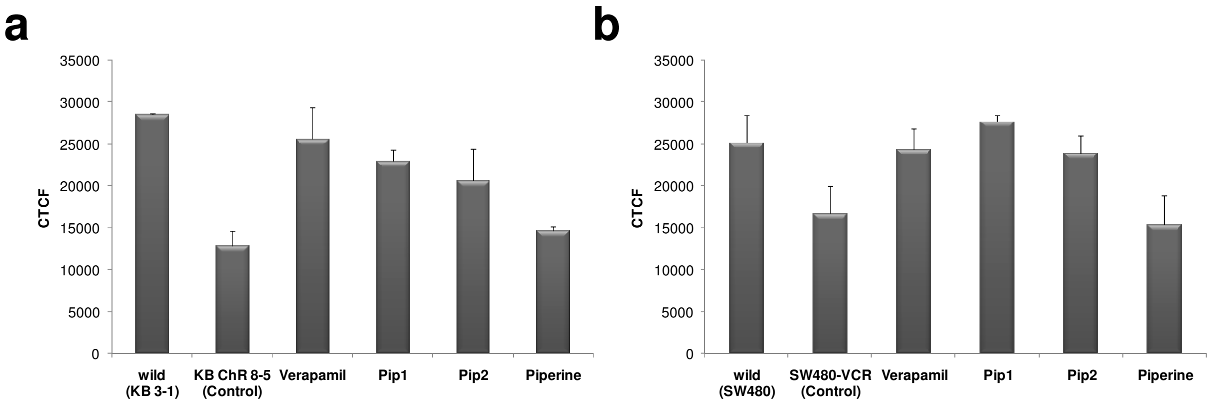

Figure S14: Quantification of Rho123 fluorescence in (a) parental KB 3-1 and resistant KB Ch^R 8-5 cells and (b) parental SW480 and resistant SW480-VCR cells using ImageJ. The corrected total cell fluorescence (CTCF) was calculated using the formula: integrated density – (area of selected cell × mean fluorescence of background readings). The analysis was performed on at least 25 cells, n = 2.

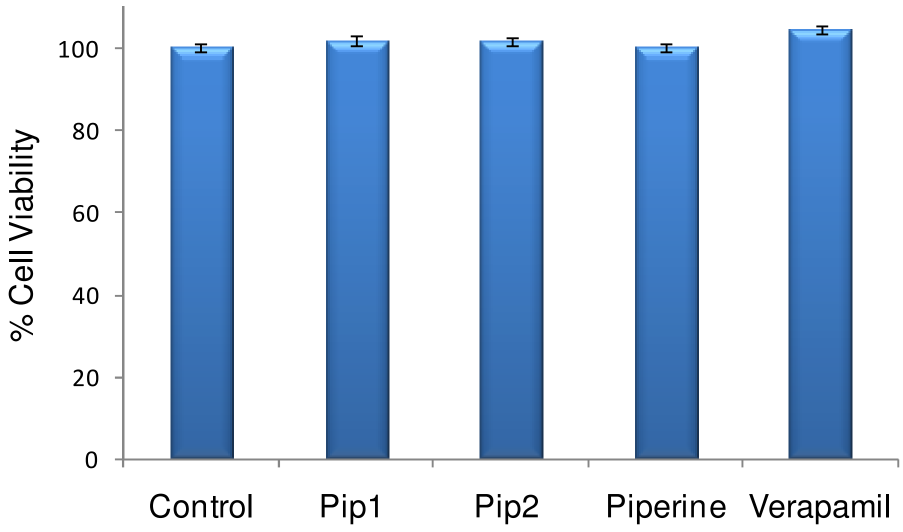

Figure S15: Cytotoxicity of Pip1, Pip2, piperine, and verapamil on non-cancer human embryonic kidney cells (HEK 293) at 4 µM concentration. Error bars represent the mean ± SEM of three independent experiments, each done in triplicates.

References

1 Loo, T. W. & Clarke, D. M. Superfolding of the partially unfolded core-glycosylatedintermediate of human P-glycoprotein into the mature enzyme is promoted by substrate-induced transmembrane domain interactions. J Biol Chem 273, 14671-14674 (1998).
